# Detecting and tracking drift in quantum information processors

**DOI:** 10.1038/s41467-020-19074-4

**Published:** 2020-10-26

**Authors:** Timothy Proctor, Melissa Revelle, Erik Nielsen, Kenneth Rudinger, Daniel Lobser, Peter Maunz, Robin Blume-Kohout, Kevin Young

**Affiliations:** 1 Quantum Performance Laboratory, Sandia National Laboratories, Albuquerque, NM 87185 USA; 2 Quantum Performance Laboratory, Sandia National Laboratories, Livermore, CA 94550 USA; 3 Sandia National Laboratories, Albuquerque, NM 87185 USA

**Keywords:** Statistics, Information theory and computation, Quantum information, Qubits

## Abstract

If quantum information processors are to fulfill their potential, the diverse errors that affect them must be understood and suppressed. But errors typically fluctuate over time, and the most widely used tools for characterizing them assume static error modes and rates. This mismatch can cause unheralded failures, misidentified error modes, and wasted experimental effort. Here, we demonstrate a spectral analysis technique for resolving time dependence in quantum processors. Our method is fast, simple, and statistically sound. It can be applied to time-series data from any quantum processor experiment. We use data from simulations and trapped-ion qubit experiments to show how our method can resolve time dependence when applied to popular characterization protocols, including randomized benchmarking, gate set tomography, and Ramsey spectroscopy. In the experiments, we detect instability and localize its source, implement drift control techniques to compensate for this instability, and then demonstrate that the instability has been suppressed.

## Abstract (web summary)

Time-dependent errors are one of the main obstacles to fully-fledged quantum information processing. Here, the authors develop a general methodology to monitor time-dependent errors, which could be used to make other characterisation protocols time-resolved, and demonstrate it on a trapped-ion qubit.

## Introduction

Recent years have seen rapid advances in quantum information processors (QIPs). Testbed processors containing tens of qubits are becoming commonplace^1–4^ and error rates are being steadily suppressed^1,5^, fueling optimism that useful quantum computations will soon be performed. Improved theories and models of the types and causes of errors in QIPs have played a crucial role in these advances. These new insights have been made possible by a range of powerful device characterization protocols^5–15^ that allow scientists to probe and study QIP behavior. But almost all of these techniques assume that the QIP is stable—that data taken over a second or an hour reflects some constant property of the processor. These methods can malfunction badly if the actual error mechanisms are time-dependent^16–22^.

Yet temporal instability in QIPs is ubiquitous^21–32^. The control fields used to drive logic gates drift^22^, *T*_1_ times can change abruptly^32^, low-frequency 1/*f*^*α*^ noise is common^24^, and laboratory equipment produces strongly oscillating noise (e.g., 50 Hz/60 Hz line noise and  ~1 Hz mechanical vibrations from refrigerator pumps). These intrinsically time-dependent error mechanisms are becoming more and more important as technological improvements suppress stable and better-understood errors. As a result, techniques to characterize QIPs with time-dependent behavior are becoming increasingly necessary.

In this article, we introduce and demonstrate a general, flexible, and powerful methodology for detecting and measuring time-dependent errors in QIPs. The core of our techniques can be applied to time-series data from any set of repeated quantum circuits—so they can be applied to most QIP experiments with only superficial adaptations—and they are sensitive to both periodic instabilities (e.g., 50 Hz/60 Hz line noise) and aperiodic instabilities (e.g., 1/*f*^*α*^ noise). This means that they can be used for routine, consistent stability analyses across QIP platforms and that they can be applied to data gathered primarily for other purposes, e.g., data from running an algorithm or error correction. Moreover, we show how to use our methods to upgrade standard characterization protocols—including randomized benchmarking (RB)^7–14^ and gate set tomography (GST)^5,6^—into time-resolved techniques. Our methods, therefore, induce a suite of general-purpose drift characterization techniques, complementing tools that focus on specific types of drift^23–26,33–43^. We demonstrate our techniques using both simulations and experiments. In our experiments, we implemented high precision, time-resolved Ramsey spectroscopy, and GST on a ^171^Yb^+^ ion qubit. We detected a small instability in the gates, isolated its source, and modified the experiment to compensate for the discovered instability. By then repeating the GST experiment on the stabilized qubit, we were able to show both improved error rates and that the drift had been suppressed.

## Results

### Instability in quantum circuits

Experiments on QIPs almost always involve choosing some quantum circuits and running them many times. The resulting data is usually recorded as counts^5–15^ for each circuit—i.e., the total number of times each outcome was observed for each circuit. Dividing these counts by the total number of trials yields frequencies that serve as good estimates of the corresponding probabilities averaged over the duration of the experiment. But if the QIP’s properties vary over that duration, then the counts do not capture all the information available in the data, and time-averaged probabilities do not faithfully describe the QIP’s behavior. The counts may then be irreconcilable with any model for the QIP that assumes that all operations (state preparations, gates, and measurements) are time-independent. This discrepancy results in failed or unreliable tomography and benchmarking experiments^16–22^.

**Fig. 1:**
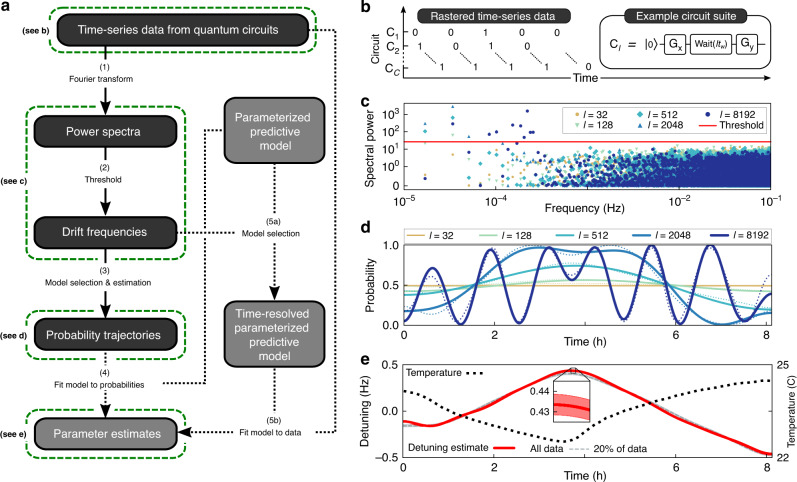
Diagnosing time-dependent errors in a quantum information processor. **a** A flowchart of our methodology for detecting and quantifying drift in a QIP, using time-series data from quantum circuits. The core steps (1–3) detect instability, identify the dominant frequencies in any drift, and estimate the circuit outcome probabilities over time. They can be applied to data from any set of quantum circuits, including data collected primarily for other purposes. Additional steps (4 and/or 5) estimate time-varying parameters (e.g., error rates) whenever a time-independent parameterized model is provided for predicting circuit outcomes. Such a model is readily available whenever the circuits are from an existing characterization technique, such as Ramsey spectroscopy or gate set tomography. **b** An example of circuits on which this technique can be implemented—Ramsey circuits with a variable wait time *lt*_w_—as well as an illustration of data obtained by “rastering” (each circuit is performed once in sequence and this sequence is repeated *N* times). **c**–**e** Results from performing these Ramsey circuits on a ^171^Yb^+^ ion qubit (*l* = 1, 2, 4, …, 8192, *t*_w_ ≈ 400 μs, *N* = 6000). **c** The power spectra observed in this experiment for selected values of *l*. Frequencies with power above the threshold almost certainly appear in the true time-dependent circuit probabilities, *p*_*l*_(*t*). **d** Estimates of the probability trajectories (unbroken lines are estimates from applying step 3 of the flowchart; dotted lines are the probabilities implied by the time-resolved detuning estimate shown in **e**). **e** The standard Ramsey model ${p}_{l}(t)=A+B\sin (2\pi l{t}_{\mathrm{w}}\Omega )$, where Ω is the qubit detuning, is promoted to a time-resolved parameterized model (step 5a) and fit to the data (step 5b) using maximum likelihood estimation, resulting in a time-resolved detuning estimate (red unbroken line). The detuning is strongly correlated with ambient laboratory temperature (black dotted line), suggesting a causal relationship that is supported by further experiments (see the main text). The detuning can still be estimated to high precision using only 20% of the data (gray dashed line), which demonstrates that our techniques could be used for high precision, targeted drift tracking while also running application circuits. The shaded areas are 2*σ* (~95%) confidence regions.

Time-resolved analysis of the data from any set of circuits can be enabled by simply recording the observed outcomes (clicks) for each circuit in sequence, rather than aggregating this sequence into counts. We call the sequence of outcomes **x** = (*x*_1_, *x*_2_, …, *x*_*N*_) obtained at *N* data collection times *t*_1_, *t*_2_, …, *t*_*N*_ a “clickstream.” There is one clickstream for each circuit. We focus on circuits with binary 0/1 outcomes (see Supplementary Note 1 for discussion of the general case), and on data obtained by “rastering” through the circuits. Rastering means running each circuit once in sequence, then repeating that process until we have accumulated *N* clicks per circuit (Fig. 1b). Under these conditions, the clickstream associated with each circuit is a string of bits, at successive times, each of which is sampled from a probability distribution over {0, 1} that may vary with time. If this probability distribution does vary over time, then we say that the circuit is temporally unstable. In this article we present methods for detecting and quantifying temporal instability, using clickstream data from any circuits, which are summarized in the flowchart of Fig. 1a.

Our methodology is based on transforming the data to the frequency domain and then thresholding the resultant power spectra. From this foundation, we generate a hierarchy of outputs: (1) yes/no instability detection; (2) a set of drift frequencies; (3) estimates of the circuit probability trajectories; and (4) estimates of time-resolved parameters in a device model. To motivate this strategy, we first highlight some unusual aspects of this data analysis problem.

Formally, a clickstream **x** is a single draw from a vector of independent Bernoulli (coin) random variables **X** = (*X*_1_, *X*_2_, …, *X*_*N*_) with biases **p** = (*p*_1_, *p*_2_, …, *p*_*N*_). Here *p*_*i*_ = *p*(*t*_*i*_) is the instantaneous probability to obtain 1 at the *i*th repetition time of the circuit, and *p*(⋅) is the continuous-time probability trajectory. The naive strategy for quantifying instability is to estimate **p** from **x** assuming nothing about its form. However, **p** consists of *N* independent probabilities and there are only *N* bits from which to estimate them, so this strategy is flawed. The best fit is always **p** = **x**, which is a probability jumping between 0 and 1, even if the data seems typical of draws from a fixed coin. This is overfitting.

To avoid overfitting, we must assume that **p** is within some relatively small subset of all possible probability traces. Common causes of time variation in QIPs are not restricted to any particular portion of the frequency spectrum, but they are typically sparse in the frequency domain, i.e., their power is concentrated into a small range of frequencies. For example, step changes and 1/*f*^*α*^ noise have power concentrated at low frequencies, while 50 Hz/60 Hz line noise has an isolated peak, perhaps accompanied by harmonics. Broad-spectrum noise does appear in QIP systems, but because it has an approximately flat spectrum, it acts like white noise—which produces uncorrelated stochastic errors that are accurately described by time-independent models. So, we model variations as sparse in the frequency domain, but otherwise arbitrary. Note that we do not make any other assumptions about *p*(*t*). We do not assume that it is sampled from a stationary stochastic process, or that the underlying physical process is, e.g., strongly periodic, deterministic, or stochastic.

### Detecting instability

The expected value of a clickstream is the probability trajectory, and this also holds in the frequency domain. That is, ${\mathbb{E}}[\tilde{{\bf{X}}}]=\tilde{{\bf{p}}}$, where ${\mathbb{E}}[\cdot ]$ is the expectation value and $\tilde{{\bf{v}}}$ denotes the Fourier transform of the vector **v** (see the Methods for the particular transform that we use). In the time domain, each *x*_*i*_ is a very low-precision estimate of *p*_*i*_. In the frequency domain, each ${\tilde{x}}_{\omega }$ is the weighted sum of *N* bits, so the strong, independent shot noise inherent in each bit is largely averaged out and any non-zero ${\tilde{p}}_{\omega }$ is highlighted. Of course, simply converting to the frequency domain cannot reduce the total amount of shot noise in the data. To actually suppress noise we need a principled method for deciding when a data mode ${\tilde{x}}_{\omega }$ is small enough to be consistent with ${\tilde{p}}_{\omega }=0$. One option is to use a regularized estimator inspired by compressed sensing^44^. But we take a different route, as this problem naturally fits within the flexible and transparent framework of statistical hypothesis testing^45,46^.

We start from the null hypothesis that all the probabilities are constant, i.e., ${\tilde{p}}_{\omega }=0$ for every *ω* > 0 and every circuit. Then, for each *ω* and each circuit, we conclude that $| {\tilde{p}}_{\omega }|\; > \; 0$ only if $| {\tilde{x}}_{\omega }|$ is so large that it is inconsistent with the null hypothesis at a pre-specified significance level *α*. If we standardize **x**, by subtracting its mean and dividing by its variance, then this procedure becomes particularly transparent: if the probability trace is constant, then the marginal distribution of each Fourier component ${\tilde{X}}_{\omega }$ for *ω* > 0 is approximately normal, and so its power $| {\tilde{X}}_{\omega }{| }^{2}$ is ${\chi }_{1}^{2}$ distributed. So if $| {\tilde{x}}_{\omega }{| }^{2}$ is larger than the (1 − *α*)-percentile of a ${\chi }_{1}^{2}$ distribution, then it is inconsistent with ${\tilde{p}}_{\omega }=0$. To test at every frequency in every circuit requires many hypothesis tests. Using standard techniques^45,46^, we set an *α*-significance power threshold such that the probability of falsely concluding that $| {\tilde{p}}_{\omega }|\; > \; 0$ at any frequency and for any circuit is at most *α* (i.e., we seek strong control of the family-wise error rate; see Supplementary Note 1).

We now demonstrate this drift detection method with data from a Ramsey experiment on a ^171^Yb^+^ ion qubit suspended above a linear surface-electrode trap^5^ and controlled using resonant microwaves. Shown in Fig. 1b, these circuits consist of preparing the qubit on the $\hat{x}$ axis of the Bloch sphere, waiting for a time *lt*_w_ (*l* = 1, 2, 4, …, 8192, *t*_w_ ≈ 400 μs), and measuring along the $\hat{y}$ axis. We performed 6000 rasters through these circuits, over ~8 h. A representative subset of the power spectra for these data are shown in Fig. 1c, as well as the *α*-significance threshold for *α* = 5%. The spectra for circuits containing long wait times exhibit power above the detection threshold, so instability was detected. These data are inconsistent with constant probabilities. Ramsey circuits are predominantly sensitive to phase accumulation, caused by detuning between the qubit and the control field frequencies, so it is reasonable to assume that it is this detuning that is drifting. The detected frequencies range from the lowest Fourier basis frequency for this experiment duration, which is ~15 μHz, up to ~250 μHz. The largest power is more than 1700 standard deviations above the expected value under the null hypothesis, which is overwhelming evidence of temporal instability.

### Quantifying instability

Statistically significant evidence in data for time-varying probabilities does not directly imply anything about the scale of the detected instability. For instance, even the weakest periodic drift will be detected with enough data. We can quantify instability in any circuit by the size of the variations in its outcome probabilities. We can measure this size by estimating the probability trajectory **p** for each circuit (step 3, Fig. 1a). As noted above, the unregularized best-fit estimate of **p** is the observed bit-string **x**, which is overfitting. To regularize this estimate, we use model selection. Specifically, we select the time-resolved parameterized model *p*(*t*) = *γ*_0_ + ∑_*k*_*γ*_*k*_*f*_*k*_(*t*), where *f*_*k*_(*t*) is the *k*th basis function of the Fourier transform, the summation is over those frequencies with power above the threshold in the power spectrum, and the *γ*_*k*_ are parameters constrained only so that each *p*(*t*) is a valid probability. We can then fit this model to the clickstream for the corresponding circuit, using any standard data fitting routine, e.g., maximum likelihood estimation.

Estimates of the time-resolved probabilities for the Ramsey experiment are shown in Fig. 1d (unbroken lines). Probability traces are sufficient for heuristic reasoning about the type and size of the errors, and this is often adequate for practical debugging purposes. For example, these probability trajectories strongly suggest that the qubit detuning is slowly drifting. To draw more rigorous conclusions, we can implement time-resolved parameter estimation.

### Time-resolved benchmarking and tomography

The techniques presented so far provide a foundation for time-resolved parameter estimation, e.g., time-resolved estimation of gate error rates, rotation angles, or process matrices. We introduce two complementary approaches, which we refer to as “non-intrusive” and “intrusive”, that can add time resolution to any benchmarking or tomography protocol. The non-intrusive approach is to replace counts data with instantaneous probability estimates in existing benchmarking/tomography analyses (step 4, Fig. 1a). It is non-intrusive because it does not require modifications to existing analysis codes. In contrast, the intrusive approach builds an explicitly time-resolved model and fits its parameters to the time-series data. We now detail and demonstrate these two techniques.

All standard characterization protocols, including all forms of tomography^5,6^ and RB^7–14^, are founded on some time-independent parameterized model that describes the outcome probabilities for the circuits in the experiment, or a coarse-graining of them (e.g., mean survival probabilities in RB). When analyzing data from these experiments, the counts data from these circuits are fed into an analysis tool that estimates the model parameters, which we denote {*γ*_*i*_}. To upgrade such a protocol using the non-intrusive method, we: (i) use the spectral analysis tools above to construct time-resolved estimates of the probabilities; (ii) for a given time, *t*_*j*_, input the estimated probabilities directly into the analysis tool in place of frequencies; (iii) recover an estimate of the model parameters, {*γ*_*i*_(*t*_*i*_)} at that time; and (iv) repeat for all times of interest {*t*_*j*_}. This non-intrusive approach is simple, but statistically *ad hoc*.

The intrusive approach permits statistical rigor at the cost of more complex analysis. It consists of (i) selecting an appropriate time-resolved model for the protocol and (ii) fitting that model to the time-series data (steps 5a-5b, Fig. 1a). In the model selection step, we expand each model parameter *γ* into a sum of Fourier components: *γ* → *γ*_0_ + ∑_*ω*_*γ*_*ω*_*f*_*ω*_(*t*), where the *γ*_*ω*_ are real-valued amplitudes, and the summation is over some set of non-zero frequencies. This set of frequencies can vary from one parameter to another and may be empty if the parameter in question appears to be constant. To choose these expansions we need to understand how any drift frequencies in the model parameters would manifest in the circuit probability trajectories, and thus in the data.

To demonstrate the intrusive approach, we return to the Ramsey experiment. In the absence of drift the probability of “1” in a Ramsey circuit with a wait time of *lt*_w_ is ${p}_{l}=A+B\exp (-l/{l}_{0})\sin (2\pi l{t}_{\textrm{w}}\Omega )$, where Ω is the detuning between the qubit and the control field, 1/*l*_0_ is the rate of decoherence per idle, and *A*, *B* ≈ 1/2 account for any state preparation and measurement errors. In our Ramsey experiment, the probability trace estimates shown in Fig. 1c suggest that the state preparation, measurement, and decoherence error rates are approximately time-independent, as the contrast is constant over time. So we define a time-resolved model that expands only Ω into a time-dependent summation:

1 $${p}_{l}(t)=A+B\exp (-l/{l}_{0})\sin (2\pi l{t}_{\textrm{w}}\Omega (t)),$$

where Ω(*t*) = *γ*_0_ + ∑_*ω*_*γ*_*ω*_*f*_*ω*_(*t*). To select the set of frequencies in the summation, we observe that the dependence of the circuit probabilities on Ω is approximately linear for small *l* (e.g., expand Eq. (1) around *lt*_w_Ω(*t*) ≈ 0). Therefore, the oscillation frequencies in the model parameters necessarily appear in the circuit probabilities. So in our expansion of Ω, we include all 13 frequencies detected in the circuit probabilities (i.e., the ones with power above the threshold in Fig. 1c). The circuit probabilities will also contain sums, differences, and harmonics of the frequencies in the true Ω—Fig. 1d shows clearly that the phase is wrapping around the Bloch sphere in the circuits with the longest wait times (*l* ≥ 2048), so these harmonic contributions will be significant in our data. Therefore, this frequency selection strategy could result in erroneously including some of these harmonics in our model. We check for this using standard information-theoretic criteria^47^ and then discard any frequencies that should not be in the model (Supplementary Note 2). This avoids overfitting the data. Once the model is selected, we have a time-resolved parameterized model that we can directly fit to the time-series data. We do this with maximum likelihood estimation.

Figure 1e shows the estimated qubit detuning Ω(*t*) over time. It varies slowly between approximately  −0.5 and +0.5 Hz. The detuning is correlated with an ancillary measurement of the ambient laboratory temperature (the Spearman correlation coefficient magnitude is 0.92), which fluctuates by  ~1.5 °C over the course of the experiment. This suggests that temperature fluctuations are causing the drift in the qubit detuning (this conclusion is supported by further experiments: see later and the Methods). The detuning has been estimated to high precision, as highlighted by the 2*σ* confidence regions in Fig. 1e. As with all standard confidence regions, these are in-model uncertainties, i.e., they do not account for any inadequacies in the model selection. However, we can confirm that the estimated detuning is reasonably consistent with the data by comparing the *p*_*l*_(*t*) predicted by the estimated model (dotted lines, Fig. 1d) with the model-independent probability estimates obtained earlier (unbroken lines, Fig. 1d). These probabilities are in close agreement.

### Demonstration on simulated data

RB^7–14^ and GST^5,6^ are two of the most popular methods for characterizing a QIP. Both methods are robust to state preparation and measurement errors; RB is fast and simple, whereas GST provides detailed diagnostic information about the types of errors afflicting the QIP. We now demonstrate time-resolved RB and GST on simulated data, using the general methodology introduced above. The number of circuits and circuit repetitions in these simulated experiments are in line with standard practice for these techniques, so they demonstrate that our techniques can be applied to RB and GST without additional experimental effort.

**Fig. 2:**
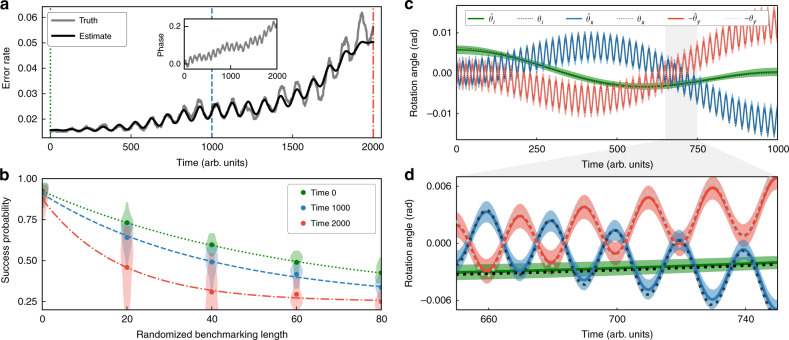
Time-resolved benchmarking & tomography on simulated data. **a**, **b** Time-resolved RB on simulated data for gates with time-dependent phase errors. **a** Inset: the simulated phase error over time. Main plot: the true, time-dependent RB error rate (*r*) versus time (grey line) and a time-resolved estimate obtained by applying our techniques to simulated data (black line). **b** Instantaneous average-over-circuits (points) and per-circuit (distributions) success probabilities at each circuit length, estimated by applying our spectral analysis techniques to the simulated time-series data, and fits to an exponential (curves), for the three times denoted by the vertical lines in **a**. Each instantaneous estimate of *r*, shown in **a**, is a rescaling of the decay rate of the exponential fit at that time. **c**, **d** Time-resolved GST on simulated data, for three gates *G*_*i*_, *G*_*x*_, and *G*_*y*_ that are subject to time-dependent coherent errors around the $\hat{z}$, $\hat{x}$, and $\hat{y}$ axes, respectively, by angles *θ*_*i*_, *θ*_*x*_, and *θ*_*y*_. The estimates of these rotation angles (denoted ${\hat{\theta }}_{{\mathsf{i}}}$, ${\hat{\theta }}_{{{x}}}$ and ${\hat{\theta }}_{{{y}}}$) track the true values closely. The shaded areas are 2*σ* (~95%) confidence regions.

We simulated data from 2000 rasters through 100 randomly sampled RB circuits^7–9^ on two qubits. The error model consisted of 1% depolarization on each qubit and a time-dependent coherent $\hat{z}$-rotation that is shown in the inset of Fig. 2a (see Supplementary Note 2 for details). The general instability analysis was implemented on this simulated data, after converting the 4-outcome data to the standard “success”/“fail” format of RB. This analysis yielded a time-dependent success probability for each circuit. Following our non-intrusive framework, instantaneous success probabilities at each time of interest were then fed into the standard RB data analysis (fitting an exponential) as shown for three times in Fig. 2b. The instantaneous RB error rate estimate is then (up to a constant^9^) the decay rate of the fitted exponential at that time. The resultant time-resolved RB error rate is shown in Fig. 2a. It closely tracks the true error rate.

GST is a method for high-precision tomographic reconstruction of a set of time-independent gates, state preparations, and measurements^5,6^. We consider GST on a gate set comprising of standard $\hat{z}$-axis preparation and measurement, and three gates *G*_*x*_, *G*_*y*_, and *G*_*i*_. Here *G*_*x/y*_ are *π*/2 rotations around the $\hat{x}/\hat{y}$ axes and *G*_*i*_ is the idle gate. The GST circuits have the form ${{\mathsf{S}}}_{\text{prep}}{{\mathsf{S}}}_{\,\text{germ}\,}^{k}{{\mathsf{S}}}_{\text{meas}}$ (circuits are written in operation order where the leftmost operation occurs first). In this circuit: S_prep_ and S_meas_ are each one of six short sequences chosen to generate tomographically complete state preparations and measurements; S_germ_ is one of twelve short “germ” sequences, chosen so that powers (repetitions) of these germs amplify all coherent, stochastic and amplitude-damping errors; *k* runs over an approximately logarithmically spaced set of integers, given by *k* = ⌊*L*/∣S_germ_∣⌋ where ∣S_germ_∣ is the length of the germ and $L={2}^{0},{2}^{1},{2}^{2},\ldots ,{L}_{\max }$ for some maximum germ power ${L}_{\max }$.

We simulated data from 1000 rasters through these GST circuits (with ${L}_{\max }=128$). The error model consisted of 0.1% depolarization on each gate. Additionally, *G*_*x*_ and *G*_*y*_ are subject to over/under-rotation errors that oscillate both quickly and slowly, while *G*_*i*_ is subject to slowly varying $\hat{z}$-axis coherent errors. We used our intrusive approach to time-resolved tomography: the general instability analysis was implemented on this simulated data, the results were used to select a time-resolved model for the gates, and this model was then fit to the time-series data using maximum likelihood estimation (see Supplementary Note 2 for details). The resulting time-resolved estimates of the gate rotation angles are shown in Fig. 2c, d. The estimates closely track the true values.

### Demonstration on experimental data

Having verified that our methods are compatible with data from GST circuits, we now demonstrate time-resolved GST on two sets of experimental data, using the three gates *G*_*x*_, *G*_*y*_, and G_*i*_. These experiments comprehensively quantify the stability of our ^171^Yb^+^ qubit, because the GST circuits are tomographically complete and they amplify all standard types of error in the gates. The *G*_*x*_ and *G*_*y*_ gates were implemented with BB1 compensated pulses^48,49^, and *G*_*i*_ was implemented with a dynamical decoupling *X*_*π*_*Y*_*π*_*X*_*π*_*Y*_*π*_ sequence^50^, where *X*_*π*_ and *Y*_*π*_ represent *π* pulses around the $\hat{x}$ and $\hat{y}$ axes. The first round of data collection included the GST circuits to a maximum germ power of *L*_max_ = 2048 (resulting in 3889 circuits). These circuits were rastered 300 times over ~5.5 h.

**Fig. 3:**
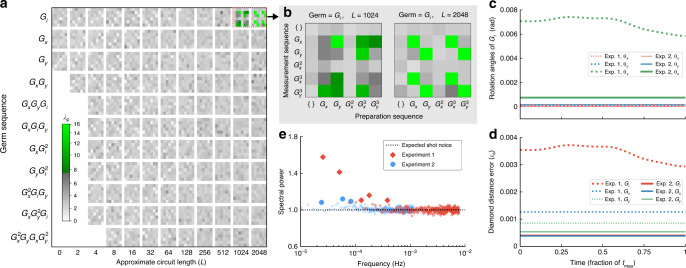
Measuring qubit stability using time-resolved GST. The results of two time-resolved GST experiments using the gates *G*_i_, *G*_*x*_, and *G*_*y*_, with drift compensation added for the second experiment. **a**, **b** The evidence for instability in each circuit in the first experiment, quantified by ${\lambda }_{{\rm{p}}}=-{\mathrm{log}\,}_{10}({\rm{p}})$ where *p* is the *p*-value of the largest power in the spectrum for that circuit. A pixel is colored green when *λ*_*p*_ is large enough to be 5% statistically significant, otherwise, it is greyscale. Each circuit consists of repeating a “germ” sequence in between six initialization and pre-measurement sequences. The data is arranged by germ and approximate circuit length *L*, and then separated into the 6 × 6 different preparation and measurement sequence pairs, as shown on the axes of B (“{}” denotes the null sequence). Only long circuits containing repeated applications of *G*_i_ exhibit evidence of drift. In the second experiment, none of the *λ*_*p*_ are statistically significant (data are not shown). **c**, **d** Time-resolved tomographic reconstructions of the gates in each experiment, summarized by the diamond distance error of each gate, and the decomposition of the coherent errors in *G*_i_ into rotation angles around $\hat{x}$, $\hat{y}$ and $\hat{z}$ (${t}_{\max }\approx 5.5$ h and ${t}_{\max }\approx 40$ h for the first and second experiment, respectively). **e** The power spectrum for each experiment obtained by averaging the individual power spectra for the different circuits, with filled points denoting power above the 5% significance thresholds (the thresholds are not shown).

Figure 3a, b summarizes the results of our general instability assessment on this data, using a representation that is tailored to GST circuits. Each pixel in this plot corresponds to a single circuit and summarizes the evidence for instability by ${\lambda }_{{\mathrm{p}}}=-{\mathrm{log}\,}_{10}({\rm{p}})$, where *p* is the *p*-value of the largest power in the spectrum for that circuit (*λ*_*p*_ is 5% significant when it is above the multi-test adjusted threshold *λ*_*p*,threshold_ ≈ 7). The only circuits that displayed detectable instability are those that contain many sequential applications of *G*_*i*_. Figure 3b further narrows this down to generalized Ramsey circuits, whereby the qubit is prepared on the equator of the Bloch sphere, active idle gates are applied, and then the qubit is measured on the equator of the Bloch sphere. These circuits amplify erroneous $\hat{z}$-axis rotations in *G*_*i*_. Other GST circuits amplify all other errors, but none of those circuits exhibit detectable drift. This is conclusive evidence that the angle of these $\hat{z}$-axis rotations is varying over the course of the experiment.

The instability in *G*_*i*_ can be quantified by implementing time-resolved GST, with the $\hat{z}$-axis error in *G*_*i*_ expanded into a summation of Fourier coefficients (see Supplementary Note 2 for details). The results are summarized in Fig. 3c, d (dotted lines). Figure 3d shows the diamond distance error rate (*ϵ*_♢_)^51^ in the three gates over time. It shows that *G*_*i*_ is the worst performing gate and that the error rate of *G*_*i*_ drifts substantially over the course of the experiment (*ϵ*_♢_ varies by ~25%). The gate infidelities are an order of magnitude smaller (Supplementary Table 1). Figure 3c shows the coherent component of the *G*_*i*_ gate over time, resolved into rotation angles *θ*_*x*_, *θ*_*y*_, and *θ*_*z*_ around the three Bloch sphere axes $\hat{x}$, $\hat{y}$, and $\hat{z}$. The varying $\hat{z}$-axis component is the dominant source of error.

This first round of experiments revealed instability, so we changed the experimental setup. Changes included the addition of periodic recalibration of the microwave drive frequency, the *π*-pulse duration, and the pointing of the detection laser (details in the Methods). We then repeated this GST experiment. To increase sensitivity to any instability, we collected more data, over a longer time period, and we included longer circuits. We ran the GST circuits out to a maximum germ power of *L*_max_ = 16,384, rastering 328 times through this set of 5041 circuits over ~40 h. The purpose of running such a comprehensive experiment was to maximize sensitivity—our methods need much fewer experimental resources for useful results (see below). Repeating the above analysis on this data, we found that none of the *λ*_*p*_ were statistically significant, i.e., no instability was detected in any circuit, including circuits containing over 10^5^ sequential *G*_*i*_ gates. Again, we performed time-resolved GST. Since no time dependence was detected, this reduces to standard time-independent GST. The results are summarized in Fig. 3c, d (unbroken lines). The gate error rates have been substantially suppressed (*ϵ*_♢_ decreased by  ~10×  for *G*_*i*_), and the $\hat{z}$-axis coherent error in *G*_*i*_ reduced and stabilized. This is a comprehensive demonstration that the recalibrations are stabilizing the qubit. Furthermore, the recalibrated parameters versus time are strongly correlated with ambient laboratory temperature (see the Methods), suggesting temperature stabilization as an alternative route to qubit stabilization, and supporting the conclusions of our Ramsey experiments.

No individual circuit exhibited signs of drift in this second GST experiment, but we can also perform a collective test for instability on the clickstreams from all the circuits. In particular, we can average the per-circuit power spectra, and look for statistically significant peaks in this single spectrum. This suppresses the shot noise inherent in each individual clickstream, so it can reveal low-power drift that would otherwise be hidden in the noise (Supplemental Note 1). This average spectrum is shown for both experiments in Fig. 3e. The power at low frequencies decreases substantially from the first to the second experiment, further demonstrating that our drift compensation is stabilizing the qubit. However, there is power above the 5% significance threshold for both experiments. So there is still some residual instability after the experimental improvements. But this residual drift is no longer a significant source of errors, as demonstrated by the low and stable error rates shown in Fig. 3d.

### Experiment design

Our method is an efficient way to identify time dependence in the outcome probability distribution of any quantum circuit. In its most basic application, it can verify the stability of application or benchmarking data. No special-purpose circuits are required, as the drift detection can be applied to data that is already being taken. The analysis will then be sensitive to any drifting errors that impact this application, in proportion to their effect on the application.

As we have demonstrated, our method can also be used to create dedicated drift characterization protocols. This mode requires a carefully chosen set of quantum circuits that are sensitive to the specific parameters under study. Without a priori knowledge about what may be drifting, this circuit set should be sensitive to all of the parameters of a gate set. The GST circuits are a good choice. However, if only a few parameters are expected to drift, a smaller set of circuits sensitive only to these parameters can be used, resulting in a more efficient experiment. For example, Ramsey circuits serve as excellent probes of time variation in qubit phase rotation rates. Many of the most sensitive circuits, such as those used in GST, Ramsey spectroscopy, and robust phase estimation^15^, are periodic and extensible. These circuits achieve ${\mathcal{O}}(1/L)$ precision scaling, with *L* the maximum circuit length, up until decoherence dominates. So, by choosing a suitably large *L*, very high-precision drift tracking can be achieved, as in our experiments.

Interleaving dedicated drift characterization circuits with application circuits combines the two use cases for our methods—dedicated drift characterization and auxiliary analysis. This reduces the data acquisition rate for both the application and characterization circuits, but it directly probes whether time variation in a parametric model is correlated with drift in the outcomes of an application circuit. While this reduces sensitivity to high-frequency instabilities, much of the drift seen in the laboratory is on timescales that are long compared to the data acquisition rate. As a simple demonstration of this, we note that discarding 80% of our Ramsey data—keeping only every fifth bit for each circuit—still yields a high-precision time-resolved phase estimate, as shown in Fig. 1e (gray dashed line).

The sensitivity of our analysis depends on both the number of times a circuit is repeated (*N*) and the sampling rate (*t*_gap_). As in all signal analysis techniques, the sampling rate sets the Nyquist limit—the highest frequency the analysis is sensitive to without aliasing—while (*N* − 1)*t*_gap_ sets the lowest frequency drift that will be visible. While the sensitivity of our methods increases with more data, statistically significant results can be achieved without dedicating hours or days to data collection. For example, both the simulated GST and RB experiments (Fig. 2) used a number of circuits and repetitions consistent with standard practices. Further details relating to the sampling parameters and the analysis sensitivity are provided in Supplementary Note 3.

## Discussion

Reliable quantum computation demands stable hardware. But current standards for characterizing QIPs assume stability—they cannot verify that a QIP is stable, nor can they quantify any instabilities. This is becoming a critical concern as stable sources of errors are steadily reduced. For example, drift significantly impacted the recent tomographic experiments of Wan et al.^22^ but this was only verified using a complicated, special-purpose analysis. In this article, we have introduced a general, flexible, and powerful methodology for diagnosing instabilities in a QIP. We have applied these methods to a trapped-ion qubit, demonstrating both time-resolved phase estimation and time-resolved tomographic reconstructions of logic gates. Using these tools, we were able to identify the most unstable gate, confirm that periodic recalibration stabilized the qubit to an extent that drift is no longer a significant source of error, and isolate the probable source of the instabilities (temperature changes).

Our methods are widely-applicable, platform-independent, and do not require special-purpose experiments. This is because the core techniques are applicable to the data from any set of quantum circuits—as long as it is recorded as a time series—and the data analysis is fast and simple (speed is limited only by the fast Fourier transform). These techniques enable routine stability analysis on data gathered primarily for other purposes, such as data from algorithmic, benchmarking, or error correction circuits. These techniques are even applicable outside of the context of quantum computing—they could be used for time-resolved quantum sensing. We have incorporated these tools into an open-source software package^52,53^, making it easy to check any time-series QIP data for signs of instability. Because of the disastrous impact of drift on characterization protocols^16–22^, its largely unknown impact on QIP applications, and the minimal overhead required to implement our methods, we hope to see this analysis broadly and quickly adopted.

## Methods

### Experiment details

We trap a single ^171^Yb^+^ ion  ~34 μm above a Sandia multi-layer surface ion trap with integrated microwave antennae, shown in Supplementary Fig. 1. The radial trapping potential is formed with 170 V of rf-drive at 88 MHz; the axial field is generated by up to 2 V on the segmented dc control electrodes. This yields secular trap frequencies of 0.7, 5, and 5.5 for the axial and radial modes, respectively. An electromagnetic coil aligned with its axis perpendicular to the trap surface creates the quantization field of ~5 G at the ion. The field magnitude is calibrated using the qubit transition frequency, which has a second-order dependence on the magnetic field of *f* = 12.642 812 118 GHz + 310.8*B*^2^ Hz, where *B* is the externally applied magnetic field in Gauss^54^. The qubit is encoded in the hyperfine clock states of the ^2^S_1/2_ ground state of ^171^Yb^+^, with logical 0 and 1 defined as $\left|F=0,{m}_{F}=0\right\rangle$ and $\left|F=1,{m}_{F}=0\right\rangle$, respectively.

Each run of a quantum circuit consists of four steps: cooling the ion, preparing the input state, performing the gates, and then measuring the ion. First, using an adaptive length Doppler cooling scheme, we verify the presence of the ion. The ion is Doppler cooled for 1 ms, during which fluorescence events are counted. If the number of detected photons is above a threshold (~85% of the average fluorescence observed for a cooled ion) Doppler cooling is complete, otherwise, the cooling is repeated. If the threshold is not reached after 300 repetitions, the experiment is halted to load a new ion. This ensures that an ion is present in the trap and that it is approximately the same temperature for each run. After cooling and verifying the presence of the ion, it is prepared in the $\left|F=0,{m}_{F}=0\right\rangle$ ground state using an optical pumping pulse^55^. All active gates are implemented by directly driving the 12.6428 GHz hyperfine qubit transition, using a near-field antenna integrated into the trap (Supplementary Fig. 1). The methods used for generating microwave radiation are discussed in ref. ^5^. A standard state fluorescence technique^55^ is used to measure the final state of the qubit.

The gates we use are *G*_*x*_, *G*_*y*_, and *G*_*i*_, which are *π*/2 rotations around the $\hat{x}$- and $\hat{y}$-axes, and an idle gate. The *G*_*x*_ and *G*_*y*_ gates, used in both the Ramsey and GST experiments, are implemented using BB1 pulse sequences^48,49^. The *G*_*i*_ gate, used only in the GST experiments, is a second-order compensation sequence: *G*_*i*_ = *X*_*π*_*Y*_*π*_*X*_*π*_*Y*_*π*_, where *X*_*π*_ and *Y*_*π*_ denote *π* pulses about the $\hat{x}$- and $\hat{y}$-axis, respectively^50^. To maintain a constant power on the microwave amplifier and reduce the errors from finite on/off times, active gates are performed gapless, i.e., we transition from one pulse to the next by adjusting the phase of the microwave signal without changing the amplitude of the microwave radiation. In the first GST experiment, the Rabi frequency was 119 kHz.

Changing the phase in the analog output signal takes  ~5 ns and this causes errors because the pulse sequences are performed gapless. These errors are larger for shorter *π*-pulse times. To reduce this error, in this second GST experiment the Rabi frequency was decreased to 74 kHz. To compensate for the drift that we observed in both the Ramsey experiment and the first GST experiment, in the second GST experiment we incorporated three forms of active drift control. The detection laser position was recalibrated every 45 minutes, and both the *π*-time (*τ*_*π*_) and the microwave drive frequency (*f*) were updated based on the results of interleaved calibration circuits. After every 4th circuit, a circuit consisting of a 10.5*π* pulse was performed. If the outcome was 0 (resp., 1) then 1.25 ns was added to *τ*_*π*_ (resp., subtracted from *τ*_*π*_). The applied *π*-time is *τ*_*π*_ rounded to an integer multiple of 20 ns, so only consistently bright or dark measurements result in changes of the pulse time. After every 16th circuit, a 10 ms wait Ramsey circuit was performed. If the outcome was 0 (resp., 1) 10 mHz is added to *f* (resp., subtracted from *f*).

**Fig. 4:**
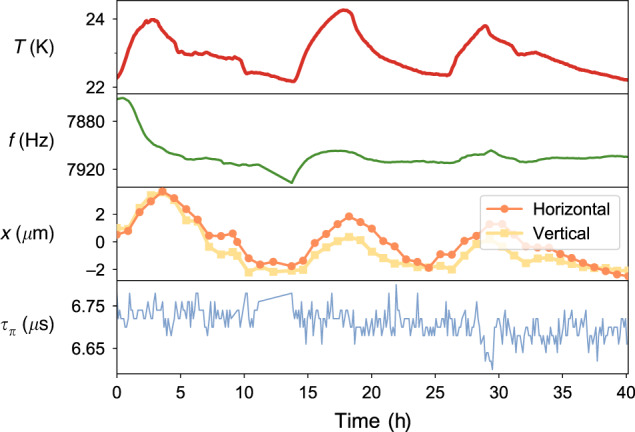
Ancillary measurements in the stabilized GST experiment. The microwave drive frequency *f*, the horizontal and vertical detection beam offsets *x*, and microwave *π*-pulse time *τ*_*π*_ were periodically recalibrated and tracked over the course of the experiment. The Spearman correlation coefficient (*ρ*) confirms that the ambient temperature *T* is well correlated with the drive frequency (*ρ* = 0.64) and the horizontal and vertical beam offsets (*ρ* = 0.86 and *ρ* = 0.84), but it is not well correlated with the *π*-pulse time (*ρ* = 0.05).

Figure 4 shows the detection beam position, *τ*_*π*_, *f*, and ambient laboratory temperature over the course of the second GST experiment. The calibrated *f* is correlated with the ambient temperature. This is consistent with the observed correlation between the ambient temperature and the estimated detuning in the Ramsey experiment (Fig. 1e). The temperature is also strongly correlated with the calibrated detection beam location points, suggesting that thermal expansion is a plausible underlying cause of the frequency shift.

### Data analysis details

To generate a power spectrum from a clickstream, we use the Type-II discrete cosine transform with an orthogonal normalization. This is the matrix *F* with elements

2 $${F}_{\omega i}=\sqrt{\frac{{2}^{1-{\delta }_{\omega ,0}}}{N}}\cos \left(\frac{\omega \pi }{N}\left(i+\frac{1}{2}\right)\right),$$

where *ω*, *i* = 0, …, *N* − 1^56^. However, note that the exact transform used is not important: we only require that *F* is an orthogonal and Fourier-like matrix (Supplementary Note 1). Our hypothesis testing is all at a statistical significance of 5% and uses a Bonferroni correction to maintain this significance when implementing many hypothesis tests (Supplementary Note 1). All data fitting uses maximum likelihood estimation, except for the *p*(*t*) estimation in the time-resolved RB simulations. In that case, we use a simple form of signal filtering (see Supplementary Note 1), so that the entire analysis chain maintains the speed and simplicity inherent to RB. When choosing between multiple time-resolved models, as in the time-resolved Ramsey tomography and GST analyses, we use the Akaike information criteria^47^ to avoid overfitting (Supplementary Note 2). Further details on these methods, and supporting theory, is provided in Supplementary Notes 1–3.

## Supplementary information

Supplementary Information

## Data Availability

All experimental and simulated data presented in this paper are available at 10.5281/zenodo.4033077.

## Code availability

The code for implementing the general drift characterization methods introduced in this paper has been incorporated into the open-source Python package pyGSTi^52,53^. The pyGSTi-based Python scripts and notebooks used for the data analysis reported in this paper are available at 10.5281/zenodo.4033077.
