# Supplementary material for: Detecting and tracking drift in quantum information processors
Source: Nat Commun. 2020 Oct 26;11:5396. doi: 10.1038/s41467-020-19074-4 (PMC7588494; doi:10.1038/s41467-020-19074-4)
Supplement: Supplementary file 1 — Supplementary Information [file 41467_2020_19074_MOESM1_ESM.pdf]

Supplementary Information

Detecting and tracking drift in quantum information processors

Proctor *et al.*

SUPPLEMENTARY NOTE 1 — DRIFT CHARACTERIZATION USING TIME-SERIES DATA

Here we provide supporting theory and information for the methods that we propose for detecting and characterizing instability in the outcome distribution of arbitrary quantum circuits. This is the methods summarized in steps 1-3 of the flowchart in Fig. 1 of the main text.

A. Power spectra

To begin, we detail how a clickstream is transformed into the frequency domain and then converted into a power spectrum (step 1 in the flowchart of Fig. 1A of the main text). To transform into the frequency domain we apply a Fourier-like transform F to rescaled data. All of the data analysis in this article uses the Type-II discrete cosine transform (DCT), with an orthogonal normalization. It is define by the matrix $F^{(\text{DCT})}$ with the elements

$$F^{(\text{DCT})}_{\omega i} = \sqrt{\frac{2^{1-\delta_{\omega,0}}}{N}} \cos\left(\frac{\omega\pi}{N}\left(i+\frac{1}{2}\right)\right), \tag{1}$$

where $\omega, i = 0, \ldots, N-1$ [1, 2]. The Type-II DCT typically results in a sparser representation of signals with differing values at the boundaries than many of the alternative Fourier-like transforms [1, 2]. Functions of this sort are typical in our problem, and so this is our motivation for using this transform.

Although all our data analysis uses the Type-II DCT, our methods and our theory (except where stated) are all directly applicable for any transform matrix F that satisfies the following criteria: (i) F is orthogonal, (ii) every matrix element of F satisfies $F^2_{\omega i} \leq b_F/N$ for a small constant b_F, and (iii) the top row of F is proportional to the vector of all ones. This includes, *e.g.*, sine transforms, other cosine transforms, and the Walsh-Hadamard transform [1–3] (all with the appropriate normalizations). Note that the standard discrete Fourier transform does not satisfy these requirements (with an appropriate normalization it is unitary, but not orthogonal). However, the methods described here are compatible with the discrete Fourier transform as long as some minor adjustments are made to guarantee that, *e.g.*, the probability trace estimates are real. Throughout the following theory, we refer to the indices of the F-domain vector as frequencies, but note that (i) when we report frequencies for experimental data these indices have been converted to Hertz, and (ii) for a generic transformation an index should only be interpreted as indicating the corresponding basis function of F, and it may or may not constitute a frequency.

As discussed in the main text, the motivation for using spectral analysis is that many physically plausible $p(t)$ are sparse in the frequency domain. But note that the relevant quantity to the data analysis is the discrete-time probability trajectory $\mathbf{p}$, where $p_i = p(t_i)$ and t_i is the ith data collection time for the circuit in question. Moreover, as we are concerned with deviations from time-independence, it is the "signal" vector

$$\mathbf{s} = \mathbf{p} - \bar{p}\mathbf{1}, \tag{2}$$

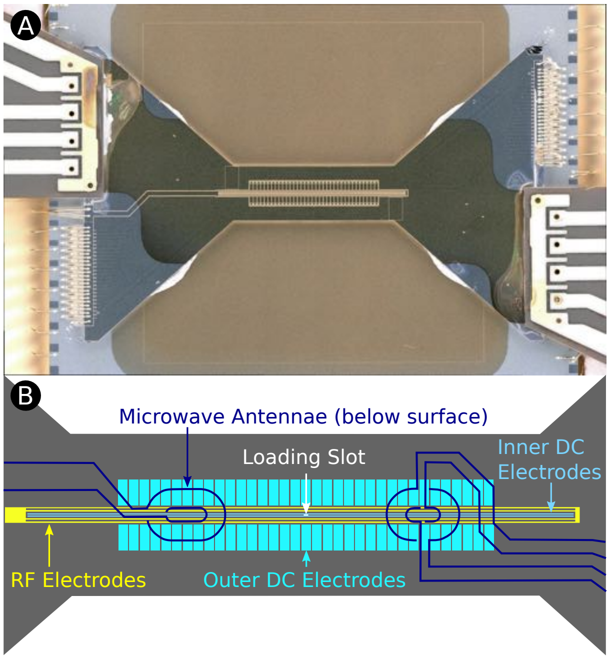

Supplementary Figure 1. **The Sandia ion trap**. A $^{171}Yb^{+}$ ion is loaded in the center of the trap, by photo-ionizing neutral ytterbium vapor introduced through the loading slot in the device. It is then shuttled to the center of the microwave antenna on the left. **A.** Flex cable microwave waveguides (upper left and lower right) are solder-die attached to the trap die to circumvent the trap package. This limits unintended impedance changes and losses, and optimizes the microwave power reaching the antenna. **B.** A schematic of the trap showing the antennae locations with respect to the dc control and rf electrodes. The leads for the microwave antennae attach directly to the flex cable waveguides. The antennae are located on the lowest metal layer underneath the trap electrodes. While the generated magnetic field is attenuated by the metal layers above it, the gaps between electrodes make this design viable and the magnetic field above the trap is about half of the field strength generated if the antennae would not be covered by trap electrodes. In the Ramsey experiment the ion was situated at the center of the trap, above the loading slot. In both GST experiments the ion was located above the center of the left microwave antenna.

that is of most relevance, where $\bar{v}$ denotes the mean of the vector $\mathbf{v}$ and $\mathbf{1} = (1, 1, \ldots, 1)^{\text{T}}$. It is sparsity of $\tilde{\mathbf{s}}$ that is important for our data analysis. Sparsity in the frequency domain of the continuous-time probability $p(t)$ implies that $\tilde{\mathbf{s}} = F\mathbf{s}$ is a sparse vector if: (i) a sensible choice is made for the transformation F, such as the Type-II DCT, (ii) the number of sample times N is sufficiently high, and (iii) the sample times $t_0, t_1, t_2, \ldots, t_{N-1}$ for the circuit in question are spaced reasonably uniformly.

All the experiments and simulations herein consist of rastering through a set of circuits — *i.e.*, running each circuit once, and then looping through this until each circuit has been run N times. So the time between sequential runs of a circuit is exactly constant (and the same for all the circuits) in an idealized experiment. Violations of this ideal are unavoidable, but if they are small (as they are in our experiments) they only mildly degrade the effectiveness of our techniques.

This can be understood by noting that small perturbations on equally-spaced times (or a small number of erroneously long time-steps of any size) will only slightly reduce the sparsity of $\tilde{\mathbf{s}}$, for any Fourier-sparse $p(t)$. Large deviations from the equally-spaced-times ideal can substantial degrade the sensitivity of the methods we presented in the main text (although this will not cause instability to be spuriously detected). In this case, we can instead generate power spectra using a technique that explicitly takes into account the sample times. One such option is the floating-mean Lomb-Scargle periodogram [4, 5], and all our methods can be adapted to this spectral analysis technique. As there are technical differences in this case, and this method is not needed to analyze our experimental data, we do not include the details here.

Before transforming a clickstream ($\mathbf{x}$) to the frequency domain, we first subtract its mean and divide by its variance. This simplifies the statistics of the power spectrum, but the spectrum is not well-defined if the clickstream consists of only zeros or only ones (because then the variance of $\mathbf{x}$ is zero). So we define the frequency-domain amplitudes by:

$$\tilde{\mathbf{z}} = \begin{cases} \frac{F(\mathbf{x}-\bar{x}\mathbf{1})}{\sqrt{\bar{x}(1-\bar{x})}} & \text{if } 0 < \bar{x} < 1, \\ (0,1,1,\dots)^{\mathsf{T}} & \text{else.} \end{cases} \tag{3}$$

The power at frequency ω is then given by $|\tilde{z}_\omega|^2$. This convention for the constant clickstream case is convenient, particularly when averaging the power spectra from multiple clickstreams, as there are then fewer situations in which we need to explicitly take this special case into account. Moreover, $(0,1,1,\dots)$ roughly corresponds to the $N \to \infty$ limit of $\tilde{\mathbf{z}}$ for a clickstream containing a single 1 or 0 (the correspondence is not precise, because the spectrum of a delta function is not completely flat for a general transform F).

B. Statistical hypothesis testing

All of our methods are built upon statistical hypothesis testing [6, 7]. We now review the relevant aspects of this field and formulate our hypothesis testing problem. Consider some set of random variables $\{A_1, A_2, \dots\}$ with a joint distribution that is parameterized by $\theta \in \mathcal{H}$ for some space $\mathcal{H}$. A statistical hypothesis is the conjecture that $\theta \in \mathcal{H}_0 \subset \mathcal{H}$. In this article, data consists of a clickstream of N bits for each of $C \geq 1$ circuits. Generalizing the notation used in the main text so that we explicitly denote the circuit index, let $x_{c,i}$ be the bit output by the ith repetition of the cth circuit, and denote the corresponding Bernoulli random variable from which that datum is drawn by $X_{c,i}$. The set of random variables about which we state statistical hypotheses is $\{X_{c,i}\}$. The random variable $X_{c,i}$ has some unknown probability $p_{c,i}$ of taking the value 1, so the set of random variables $\{X_{c,i}\}$ is parameterized by the space

$$\mathcal{H} = \{p_{c,i} \in [0,1]\}. \tag{4}$$

Statistical hypothesis testing starts from some set of null hypotheses $\{\mathcal{H}_{0,i}\}$ and uses statistical hypothesis tests on data drawn from the random variables to attempt to reject one or more of these null hypotheses (and/or intersections of these null hypotheses). Our methods look for evidence that $|\tilde{p}_{\omega,c}| > 0$ at each non-zero frequency ω and for each circuit c. This is formalized by the set of null hypotheses $\{\mathcal{H}_{0,c,\omega}\}$ where $\mathcal{H}_{0,c,\omega}$ is the conjecture that $\tilde{p}_{c,\omega} = 0$, with $\omega = 1, 2, \dots, N-1$ and $c = 1, 2, \dots, C$. For our purposes, testing a null hypothesis consists of the following four steps: (i) pick a significance level $\alpha \in (0,1)$, with $\alpha = 5\%$ a common choice; (ii) select a test statistic Λ, which is a function from data to $\mathbb{R}$; (iii) find a threshold such that the probability of observing Λ larger than this threshold if the null hypothesis is true is at most α; (iv) collect data, evaluate Λ, and reject the null hypothesis if and only if Λ is larger than the threshold. This procedure guarantees that the null hypothesis is falsely rejected with probability at most α. Our test statistic for testing the null hypothesis $\mathcal{H}_{0,c,\omega}$ is simply the power of the normalized data at the frequency ω, *i.e.*, $|\tilde{z}_{c,\omega}|^2$.

Implementing multiple hypothesis tests that use the above procedure will typically cause the probability of falsely rejecting at least one null hypothesis — the family-wise error rate (FWER) — to increase quickly with the number of tests (for T tests FWER $\leq T\alpha$). The standard approach when implementing multiple tests is to adapt the testing procedure to, at a minimum, maintain weak control of the FWER. Weak control of the FWER means that if all the null hypotheses are true then, for some pre-specified global significance α, the probability of falsely rejecting one or more null hypotheses is at most α. It is common to choose a testing procedure that also maintains strong control of the FWER. Strong control of the FWER means that the probability of falsely rejecting one or more true null hypotheses is at most α and this holds even if one or more of the null hypotheses are false. We seek to maintain strong control of the FWER, so that whenever we reject $\mathcal{H}_{0,c,\omega}$ we can be $(1-\alpha)$ confident that ω is a frequency with a non-zero amplitude in the Fourier decomposition of $\mathbf{p}_c$.

Strong control of the FWER at a global significance of α can be maintained by implementing the ith of T tests at a local significance of αw_i for any $w_i \geq 0$ satisfying $\sum_i w_i = 1$. This is known as the (generalized) Bonferroni correction [6, 7]. The Bonferroni correction can be conservative; there are other methods for maintaining strong control of the FWER that are more powerful than the Bonferroni correction, such as the Holms procedure [6, 7]. Moreover, strong control of the FWER is not the only way to ensure "confidence" in the results — *e.g.*, we could instead choose to control the false discovery rate, which is the expected ratio of the number of falsely rejected null hypotheses to the total number of rejected null hypotheses [8]. Variants of our techniques that control the false discovery rate and/or use a different multi-test correction procedure are possible, and essentially involve setting a slightly lower power significance threshold in the analysis.

C. Statistics of the power spectra

We now derive the distribution of the power spectrum of the data, both when $p(t)$ is and is not constant. We consider the

frequency-domain data $\tilde{z}_\omega$, defined in Supplementary Eq. (3), for an arbitrary non-zero frequency ω, and we derive an approximation to the distribution of the corresponding random variable,

$$\tilde{Z}_\omega = \frac{\tilde{X}_\omega}{\sqrt{\bar{X}(1-\bar{X})}}, \tag{5}$$

where $\omega > 0$ (we are again dropping the circuit indexing, and we have ignored the edge-case of the constant clickstream instance in this equation). Note that $\bar{X} = \frac{1}{N}\sum_i X_i$ is the average of random variables, and so it is itself a random variable. In the main text we asserted that $\tilde{Z}_\omega^2$ is approximately χ_1^2 distributed whenever the probability trajectory is a constant. This is a sub-case of what we derive below, but we also study the marginal distribution of $\tilde{Z}_\omega^2$ when $\tilde{p}_\omega = 0$ but the probability trajectory is not necessarily constant (*i.e.*, there is perhaps power at one or more other non-zero frequencies). We do so in order to be able to claim that we are maintaining strong control of the FWER. By itself, the fact that $\tilde{Z}_\omega^2$ is approximately χ_1^2 distributed when the probability trajectory is constant can only be used to ensure weak control of the FWER.

The random variable $\tilde{Z}_\omega$ is rather complex to directly analyze, because it depends on both $\tilde{X}_\omega$ and $\bar{X}$. So we first make an approximation. As $\bar{X}$ is the mean of the X_i we have that $\mathbb{E}[\bar{X}] = \bar{p}$ and $\mathbb{V}[\bar{X}] = O(1/N)$, where $\mathbb{E}(A)$ and $\mathbb{V}(A)$ denote the expectation value and variance of the random variable A, respectively. Therefore

$$Z_i \approx Y_i = \frac{X_i - \bar{p}}{\sqrt{\bar{p}(1-\bar{p})}}, \tag{6}$$

where we assume that $0 < \bar{p} < 1$. So we now derive the distribution of $\tilde{Y}_\omega$, as an approximation to the distribution of $\tilde{Z}_\omega$.

The random variable $\tilde{Y}_\omega$ is the sum of N independent random variables:

$$\tilde{Y}_\omega = \sum_i F_{\omega i} Y_i. \tag{7}$$

A heuristic appeal to the central limit theorem therefore suggests that $\tilde{Y}_\omega$ is normally distributed. That is, $\tilde{Y}_\omega$ is approximately $\mathcal{N}(\mu_\omega, \nu_\omega)$ distributed for $N \gg 1$, with some mean μ_ω and variance ν_ω. Later we formally apply the central limit theorem to $\tilde{Y}_\omega$, but first we derive formulae for μ_ω and ν_ω. For any non-zero ω, μ_ω is simply a rescaling of the component of the probability trajectory at this frequency:

$$\mu_\omega \equiv \mathbb{E}(\tilde{Y}_\omega) = \sum_i \mathbb{E}\left[\frac{F_{\omega i}(X_i - \bar{p})}{\sqrt{\bar{p}(1-\bar{p})}}\right] = \frac{\tilde{p}_\omega}{\sqrt{\bar{p}(1-\bar{p})}}. \tag{8}$$

So, $\mu_\omega = 0$ under the null hypothesis that $\tilde{p}_\omega = 0$. The variance ν_ω has a more subtle dependence on the probability trajectory. By noting that $\tilde{Y}_\omega$ is a summation of independent Bernoulli random variables multiplied by constants, and by noting that the variance of a Bernoulli random variable with bias b is $b(1-b)$, we obtain

$$\nu_\omega \equiv \mathbb{V}(\tilde{Y}_\omega) = \frac{1}{\bar{p}(1-\bar{p})}\sum_i F_{\omega i}^2 p_i(1-p_i). \tag{9}$$

It then follows from the orthogonality of F that $\nu_\omega = 1$ when the probability trajectory is constant. So $\tilde{Z}_\omega$ is approximately $\mathcal{N}(0,1)$ distributed when the probability trajectory is constant. Therefore, calculating significance thresholds using the model that $\tilde{Z}_\omega^2$ is χ_1^2 distributed under the null hypothesis will maintain weak control of the FWER.

Using the relation $p_i = s_i + \bar{p}$, where $\mathbf{s}$ is the signal vector, we can rewrite the variance as

$$\nu_\omega = 1 + \frac{\Delta_\omega}{\bar{p}(1-\bar{p})}, \tag{10}$$

where

$$\Delta_\omega = \sum_{i=0}^{N-1} F_{\omega i}^2 \left(s_i[1-2\bar{p}] - s_i^2\right). \tag{11}$$

So far, F is a general Fourier-like transformation that is an orthogonal matrix (see earlier). For some transformation, such as the Walsh-Hadamard transformation (but not the DCT), $F_{\omega i}^2 = 1/N$ and so Supplementary Eq. (11) simplifies to

$$\Delta_\omega = -\frac{\|\mathbf{s}\|_2^2}{N}, \tag{12}$$

because $\sum_i s_i = 0$. Here

$$\|\mathbf{v}\|_2 = \left(\sum_i v_i^2\right)^{1/2}, \tag{13}$$

is the 2-norm. That is, the variance is upper-bounded by 1, and it decreases in proportion to the signal power per time-step (which is equal to the variance of $\mathbf{p}$). So the maximum variance over the null hypothesis space of $\tilde{p}_\omega = 0$ is $\nu_\omega = 1$. This means that modeling $\tilde{Z}_\omega^2$ with a χ_1^2 distribution is sufficient for strong control of the FWER if the power spectra are generated using, *e.g.*, the Walsh-Hadamard transformation.

For a general transformation the situation is more complicated. In particular, even with $p_\omega = 0$ it is possible to obtain $\nu_\omega > 1$ for a matrix F containing elements of magnitude greater than $1/\sqrt{N}$. Because all of our data analysis uses the Type-II DCT, defined in Supplementary Eq. (1), at this point we specialize to this transformation. In this case, simple algebra can be used to show that

$$\Delta_\omega = \frac{1}{\sqrt{2N}}\left(\tilde{s}_{2\omega}[1-2\bar{p}] - \tilde{q}_{2\omega}\right) - \frac{\|\mathbf{s}\|_2^2}{N}, \tag{14}$$

where $\mathbf{q} = (s_0^2, s_1^2, s_2^2, \dots)$ is the vector of time-domain signal powers, and we are allowing the frequency index to extend outside its range as specified in the definition of the DCT. This equation implies that the variance at ω is increased by signal power at 2ω if the oscillations are not centered on $1/2$. This can only result in a variance above 1 if the amplitude at 2ω outweighs the decrease in the variance caused by the total power per time-step in $\mathbf{s}$. So the variance at ω is maximized by a pure-tone probability trajectory oscillating between $1/2$ and 1 or 0 at a frequency of 2ω, resulting in the saturable bound $\nu_\omega \leq 1 + 1/6$. Therefore the maximal variance over the

null hypothesis space of $\tilde{p}_\omega = 0$ is $v_\omega = 7/6$. So, ignoring the two approximations made so far, there exists a probability trajectory within the null hypothesis subspace defined by $\tilde{p}_\omega = 0$ such that $\tilde{Z}_\omega$ is $\mathcal{N}(0, 7/6)$ distributed. This implies that calculating significance thresholds using a χ^2_1 does not strictly provide strong control of the FWER.

We could guarantee strong control of the FWER by calculating thresholds using a rescaled χ^2_1 distribution (or, equivalently, by normalizing the data differently). But we choose not to do this, because for a broad class of probability trajectories $v_\omega \leq 1$ for all ω. So accounting for the possibility of $v_\omega > 1$ would result in a reduction in test power — *i.e.*, higher significance thresholds — to gain statistical rigor in some edge cases. In our opinion this is not a good tradeoff, and we instead settle for maintaining weak control of the FWER and "almost" maintaining strong control of the FWER. This is true in the sense that although there are some probability trajectories for which the FWER is slightly above the desired value, (i) the increase in the FWER is small, (ii) it can only occur for extremal points in the parameter space, and (iii) it is counteracted by the conservative nature of the Bonferroni correction that we use when calculating thresholds from a χ^2_1 distribution (see later). All further statements about strong control of the FWER should be understood to implicitly contain this minor caveat.

The above reasoning was all based on the assumption that $\tilde{Y}_\omega$ is approximately $\mathcal{N}(\mu_\omega, v_\omega)$ distributed for large N. So, we now prove that the $N \to \infty$ limiting distribution of $(\tilde{Y}_\omega - \mu_\omega)/\sqrt{v_\omega}$ is $\mathcal{N}(0, 1)$, under a reasonable notion of the limit of a probability trajectory. In particular, we assume that $\epsilon_1 < \bar{p} < 1 - \epsilon_1$ and $v_\omega > \epsilon_2$ for some constants $\epsilon_1, \epsilon_2 > 0$ and all N. The proof will use Lyapunov's central limit theorem [9], which we now state. Let $\{A_1, A_2, \ldots, A_n\}$ be a sequence of independent random variables where A_i has a finite expected value μ_i and variance v_i, and define $s_n^2 = \sum_i v_i$. Then, if Lyapunov's condition

$$\lim_{n\to\infty} \frac{1}{s_n^{2+\delta}} \sum_{i=1}^{n} \mathbb{E}\left[|A_i - \mu_i|^{2+\delta}\right] = 0, \tag{15}$$

holds for some $\delta > 0$, the distribution of $A = \sum_{i=1}^n (A_i - \mu_i)/s_n$ converges to $\mathcal{N}(0, 1)$ as $N \to \infty$. The random variable $\tilde{Y}_\omega$ may be written in the format of this central limit theorem: $\tilde{Y}_\omega = \sum_i Q_{\omega i}$ where $Q_{\omega i} = F_{\omega i} Y_i$, and the expected value and variance of every $Q_{\omega i}$ is bounded (due to our assumptions). It remains to prove that Lyapunov's condition holds. We have that

$$\mathbb{E}\left[|Q_{\omega i} - \mathbb{E}[Q_{\omega i}]|^3\right] \leq O(|F_{\omega i}|^3), \tag{16}$$

because $Q_{\omega i} = F_{\omega i} Y_i$ and the moments of Y_i are bounded from above by a constant (as $\epsilon_1 < \bar{p} < 1 - \epsilon_1$). Therefore

$$\frac{1}{s_n^3} \sum_{i=0}^{N-1} \mathbb{E}\left[|Q_{\omega i} - \mathbb{E}[Q_{\omega i}]|^3\right] \leq \frac{1}{s_n^3} \sum_{i=0}^{N-1} O(|F_{\omega i}|^3), \tag{17}$$

$$= O\left(\frac{1}{\sqrt{N}}\right), \tag{18}$$

with the equality holding because $s_n^3 = v_\omega^{3/2}$ and $v_\omega > \epsilon_2$. So Lyapunov's condition holds for $\delta = 1$.

In addition to performing hypothesis testing on the power spectrum for each circuit, we also test the single power spectrum obtained by averaging over the power spectra for the different circuits, *e.g.*, see Fig. 3 in the main text. The power at frequency ω in this spectrum is given by

$$\tilde{z}^2_{\text{avg},\omega} = \frac{1}{C} \sum_{c=1}^{C} \tilde{z}^2_{c,\omega}, \tag{19}$$

where C is the number of circuits. The clickstreams for different circuits are independent, and so the corresponding random variable $\tilde{Z}^2_{\text{avg},\omega}$ is the average of independent random variables that are approximately χ^2_1 distributed under the (intersection) null hypothesis

$$\mathcal{H}_{0,\omega} = \cap_c \mathcal{H}_{0,c,\omega}, \tag{20}$$

that $\tilde{p}_{c,\omega} = 0$, for all c. Therefore $\tilde{Z}^2_{\text{avg},\omega}$ is approximately χ^2_C/C under this null hypothesis.

D. Hypothesis testing of the power spectra

Here we use the statistical model developed above to calculate the power significance threshold. We argued above that $\tilde{Z}^2_{c,\omega}$ can be modeled as a χ^2_1 random variable under the null hypothesis that $\tilde{p}_{c,\omega} = 0$ (up to minor caveats). So if we are only testing a single circuit c and a single frequency ω, then the α-significance power threshold is simply

$$T = \mathsf{CDF}_1^{-1}(1 - \alpha), \tag{21}$$

where CDF_k denotes the cumulative distribution function for the χ^2_k distribution. However, we test every circuit at every non-zero frequency, and we also test the average power spectrum. So, to maintain strong control of the FWER with a global significance of α in this set of $(N-1)(C+1)$ tests, we use a Bonferroni correction. In general, this means setting the local significance of the test on circuit c at frequency ω to $w_{c,\omega}\alpha$, and setting the local significance of the test on the average power spectrum at frequency ω to $w_\omega \alpha$ for some non-negative weightings $w_{c,\omega}$ and w_ω satisfying $\sum_\omega (w_\omega + \sum_c w_{c,\omega}) = 1$. The test weightings can be optimized for different usages.

Often it is equally important to identify any drift at every frequency and in every circuit. A one-parameter subclass of weightings that is useful under this circumstance is given by $w_{c,\omega} = (1 - w)/(C(N - 1))$ and $w_\omega = w/(N - 1)$ for some weighting factor w. This choice of weightings results in a single ω-independent significance threshold for the power in the individual power spectra for the C circuits of

$$T_{\text{individual}} = \mathsf{CDF}_1^{-1}\left[1 - \frac{(1 - w)\alpha}{(N - 1)C}\right], \tag{22}$$

and an ω-independent significance threshold for the power in the averaged spectrum of

$$T_{\text{average}} = \frac{1}{C}\mathsf{CDF}_C^{-1}\left[1 - \frac{w\alpha}{N - 1}\right]. \tag{23}$$

The weighting factor w controls the proportion of the test statistical significance that is allocated to the test on the averaged power spectrum versus the significance allocated to the individual power spectra. The averaged power spectrum does not contain information about which circuits (if any) exhibit drift, but has (under most circumstances) increased sensitivity to any drift. So the best choice for this weighting depends on the relative importance of detecting any drift versus being able to assigning it to a particular circuit. All our data analysis uses the thresholds given in Supplementary Eq. (22-23), with $\alpha = 5\%$ and $w = 1/2$, with the exception of time-resolved RB, wherein $w = 1$.

E. Estimating probability trajectories

We now explain our methods for estimating the circuit outcome probability trajectories (step 3, Fig. 1A of the main text). The methods are based on model selection, to avoid overfitting. The general form of the model that we use for a probability trajectory is:

$$p(\gamma, t) = \gamma_0 + \sum_{\omega \in \mathbb{W}} \gamma_\omega f_\omega(t), \tag{24}$$

where $f_\omega(t)$ is the ωth basis function of the chosen Fourier transform, the summation is over some set of frequencies $\mathbb{W}$ that are to be chosen using model selection, and the γ_ω are parameters constrained only so that $p(\gamma, t)$ is a valid probability at all data acquisition times (the condition that $p(\gamma, t)$ is within $[0, 1]$ for all real-valued t can cause overly severe compression of the amplitudes). In the case of the Type-II DCT the basis functions are

$$f_\omega(t) = \cos\left(\frac{\omega\pi}{N}\left[\frac{t - t_0}{t_{\text{step}}} + \frac{1}{2}\right]\right), \tag{25}$$

where, as throughout, t_i is the ith data collection time for the circuit in question, and

$$t_{\text{step}} = \frac{t_{N-1} - t_0}{N - 1}, \tag{26}$$

is the size of the time-step.

The first step is model selection to identify a set of frequencies ($\mathbb{W}$). These frequencies can be chosen using the results of the hypothesis testing on the data spectra. It is useful to allow flexibility in exactly how they are chosen from the hypothesis testing results, because this allows the techniques to be adapted to different applications. A good general-purpose option, which results in estimates with a simple statistical meaning, is to set $\mathbb{W}$ to contain those and only those frequencies that are found to be statistically significant in the power spectrum for the circuit in question. With this method we are only including frequencies that contribute a nonzero component to the true $p(t)$ with confidence $(1 - \alpha)$, where α is the chosen global significance level. All of the data analysis in this article uses this method, with the exception of the model selection in time-resolved RB (see later).

It remains to estimate the model parameters, *i.e.*, the amplitudes γ. Maximum likelihood estimation (MLE) is a statistically sound methodology. But MLE requires a numerical optimization, which can sometimes be slow. So we also provide an optimization-free method. The data amplitude $\tilde{x}_\omega$ is a well-motivated estimate of the probability trajectory amplitude at ω, since $\mathbb{E}(\tilde{X}_\omega) = \tilde{p}_\omega$. In terms of the rescaled amplitudes $\tilde{\mathbf{z}}$, this suggests setting $\gamma_\omega = S(\mathbf{x})\tilde{z}_\omega$ where $S(\mathbf{x})$ is a rescaling factor that inverts the normalization of the $\tilde{\mathbf{z}}$ amplitudes by the data standard deviation (and also rescales for the chosen normalization of the basis functions). This is intuitive but flawed: the resulting probability trajectory is not necessarily a valid probability at all times. To address this, we add a regularization to this estimator. We map each amplitude via

$$\gamma_\omega \to \gamma_\omega - \delta\, \text{sign}(\gamma_\omega), \tag{27}$$

with δ the smallest constant such that the estimated probability trajectory $p(\gamma, t)$ is within $[\epsilon, 1-\epsilon]$ at all data acquisition times t_i, for some chosen $\epsilon \geq 0$ (*e.g.*, $\epsilon = 0$ if estimates on the parameter space boundary are considered acceptable). We refer to this estimation method as the "Fourier filter," because it is a form of signal filtering. The Fourier filter returns very similar estimates to MLE under most conditions, although note that if $\epsilon = 0$ it is possible for the Fourier filter to return estimates that have a likelihood of zero.

All data analysis in this paper uses MLE, except the time-resolved RB analysis. The motivation for using the Fourier filter in time-resolved RB is that then the speed of the analysis is limited only by the fast Fourier transform and curve fitting at the times of interest. This is particularly relevant to RB, as one motivation for using RB is its speed and simplicity. Moreover, note that for all the data herein the difference between the MLE and the Fourier filter estimates is negligible.

F. Analyzing data from circuits with more than two outcomes

Throughout this article we have only considered analysis of circuits with two possible outcomes. This is sufficient for all the experiments and simulations herein. However, the techniques we present can be easily generalized to M-outcome circuits, as we now briefly outline. In this more general setting, the aim is still to detect and quantify time variation in the probability distribution over outcomes for each circuit. However M can be very large in commonly encountered circumstances, as $M = 2^n$ for an n-qubit circuit terminated with the standard measurement. Even accurate estimation of a time-independent distribution over a very large number of outcomes is infeasible with reasonable quantities of data, so it will not be possible to accurately estimate a time-dependent distribution with many outcomes. For this reasons, it is useful to allow for a coarse-graining of the outcomes into $\check{M} \leq M$ bins (*e.g.*, we can marginalize over some subset of the qubits in an n-qubit circuit or bin the outcomes into two sets). The analysis will then interrogate time-dependence in the coarse-grained probability distribution. So coarse-graining can make the analysis feasible with a reasonable amount of data, but the

analysis will be insensitive to any time dependence that disappears under the chosen coarse-graining.

Each coarse-grained outcome has a bit-string time-series associated with it, corresponding to whether or not that measurement outcome was observed at each time. A power spectrum can be calculated for each such bit-string, and exactly the same hypothesis testing performed on each such power spectrum as in the 0/1 outcomes cases (with an appropriate correction for the increased number of hypothesis tests). Moreover, the spectrum obtained by averaging the spectra for a circuit over course-grained measurement outcomes can also be tested (which will be approximately $\chi^2_{\check{M}-1}$ distributed under the null hypothesis), or it can be tested instead of the per-outcome spectra — to avoid significance dilution by implementing too many hypothesis tests. The probability trajectory estimation (step 3, Fig. 1A of the main text) then proceeds exactly as in the 0/1 outcome case, except that now the estimation is of $\check{M}$ trajectories that must sum to 1 at all times. Any time-resolved parameter estimation proceeds exactly as in the 0/1 outcome case.

SUPPLEMENTARY NOTE 2 — TIME-RESOLVED TOMOGRAPHY AND BENCHMARKING

Here we provide supporting theory and information for our time-resolved tomography and benchmarking methods. This is the methods summarized in steps 4 and 5 of the flowchart in Fig. 1 of the main text.

G. Model selection in time-resolved tomography

We now explain the model selection step in our "intrusive" approach to time-resolved tomography and benchmarking (step 5a, Fig. 1A of the main text). Our methods for time-resolved tomography and benchmarking start from a time-independent $\{\gamma_j\}$-parameterized model $\mathcal{M}$ that predicts circuit outcomes. In the intrusive approach we expand each parameter γ_j into the time-dependent form

$$\gamma_j(t) = \gamma_{j,0} + \sum_{\omega \in \mathbb{S}_j} \gamma_{j,\omega} f_\omega(t), \tag{28}$$

where the $\gamma_{j,\omega}$ are restricted only so that they satisfy any constraints on γ_j, the summation is over some set of non-zero frequencies $\mathbb{S}_j$, and j indexes all the parameters in the time-independent model. So, we obtain a new model $\mathcal{M}_{\rm tr}$ containing

$$k = \sum_j (|\mathbb{S}_j| + 1), \tag{29}$$

free parameters, where $|\mathbb{S}|$ denotes the size of the set $\mathbb{S}$. The parameterized model is entirely defined by the set $\mathbb{M} = \{\mathbb{S}_j\}$, and the model selection consists of choosing this set.

For the related problem of choosing the frequencies in the model for a probability trajectory (see above) there is a clear solution: our instability detection methods are directly testing hypotheses about the frequencies in $p(t)$, and so there is a clear statistical justification for choosing the frequencies in our model for $p(t)$ using the results of these tests. In contrast, the parameters in the model $\mathcal{M}$ do not necessarily have a simple relationship to the circuit probabilities — for an arbitrary model the relationship is entirely arbitrary. So, the relationship between the frequencies detected in the probability trajectories and the frequencies in each γ_j is also, for a general model, entirely arbitrary. However, for typical models associated with tomographic methods and circuits, under some approximation there is a simple relationship between the model parameters and the predicted probabilities.

To illustrate this, we turn to the Ramsey model with a time-dependent phase that was introduced in the main text:

$$p_l(t) = A + B\exp(-l/l_0)\sin(2\pi l t_w \Omega(t)). \tag{30}$$

If we expand around $l t_w \Omega(t) = 0$ we obtain

$$p_l(t) = A + 2\pi B\exp(-l/l_0) l t_w \Omega(t) + O((l t_w \Omega(t))^2). \tag{31}$$

So, if $l t_w \Omega(t)$ is small then the dominant frequencies in $p_l(t)$ are also necessarily frequencies in $\Omega(t)$. Equivalent expansions around the zero-error parameter values hold for the general process matrix model of GST — and most other tomographic techniques use a model that is a restriction of that in GST. Therefore, we can use the results of the hypothesis tests on the data from sufficiently short circuits to select the frequencies for the model parameters. But longer circuits are more sensitive to small variations in the model parameters, so we do not want to entirely discount that data in the model selection. To address this problem we can pick a small set of candidate models and then choose between them using the Akaike information criterion (AIC) [10]. For a parameterized model $\mathcal{M}$ with k free parameters, the AIC score for that model is

$$\mathsf{AIC}(\mathcal{M}) = 2k - 2\log(\mathcal{L}_{\max}), \tag{32}$$

where $\mathcal{L}_{\max}$ is the maximum of the likelihood function for that model. For a set of candidate parameterized models, the preferred model is the one with the minimum AIC score. Moreover, $\mathsf{AIC}(\mathcal{M}_a) - \mathsf{AIC}(\mathcal{M}_b)$ is twice the log relative likelihood of model $\mathcal{M}_b$ with respect to model $\mathcal{M}_a$, so this difference quantifies how much one model is preferred over another. This allows us to decide between multiple time-resolved models.

For the Ramsey experiment presented in the main text we select the expansion

$$\Omega(t) = \gamma_0 + \sum_{\omega \in \mathbb{W}} \gamma_\omega f_\omega(t), \tag{33}$$

where $\mathbb{W} = \{1, 2, 3, 4, 5, 6, 7, 9, 11, 12, 13, 14, 15\}$ is the set of all the frequency indices found to be significant in any of the power spectra. We then check that the data justifies including the higher frequencies in the model — because the lowest frequencies appear for circuits where l is small enough so that $l t_w \Omega(t) \approx 0$, and so they are almost certainly components in

the true $\Omega(t)$. In particular, we select the 10 candidate parameterized models defined by the frequency sets $\mathbb{W}_0 = \{1, 2, 3, 4\}$, $\mathbb{W}_1 = \mathbb{W}_0 \cup \{5\}$, $\mathbb{W}_2 = \mathbb{W}_1 \cup \{6\}$ through to $\mathbb{W}_9 = \mathbb{W}$, and compare their AIC scores, AIC_k for $k = 1, 2, \ldots, 9$. The values of $\mathsf{AIC}_k - \mathsf{AIC}_9$ are all well above zero (ranging from around 10 to around 1000). So the model containing all the frequencies is clearly favored according to the AIC. The detuning estimate reported in the main text is the MLE over this parameterized model.

There is no guarantee that $\mathbb{W}$ contains all of the frequencies that are components in the true $\Omega(t)$. We could also select some larger models to consider, but we do not do this because it would be *ad hoc*: there are $N - 14 = 5986$ remaining frequencies that we could also include, and there is no clear method for choosing the next frequency to add. We could calculate the AIC for every possible model, but this is clearly impractical. In general, within this framework of time-resolved models based on Fourier decompositions there are 2^{Nk-k} possible time-resolved models, where k is the number of parameters in the time-independent model. Moreover, this would not be a reasonable use of the AIC. When adding an erroneous parameter to a model sometimes the AIC score will decrease slightly due to random fluctuations in the data slightly favoring this model, and if we consider adding many different parameters this will almost certainly occur in at least one case.

In the main text we also presented the results of time-resolved Ramsey tomography after we had discarded 80% of our data (*i.e.*, we keep every 5th click for each circuit). This analysis followed the same procedure as the analysis on all the data. In particular, note that we implemented independent model selection, *i.e.*, we did not "cheat" by using the time-resolved model selection that was performed using all the data. In particular, using only 80% of the data resulted in a smaller model whereby $\mathbb{W} = \{1, 2, 3, 5, 6, 7, 9\}$. We therefore would not expect the two estimates of $\Omega(t)$ to be equal up to statistical uncertainty (which they are not — see Fig. 1E), as both these statistical uncertainties are standard in-model uncertainties and the two estimates are fits to different models.

H. Time-resolved RB

RB is a set of protocols for benchmarking a set of gates on n qubits [11–13]. The core protocols all follow the same general procedure:

(i) For a range of lengths m, run K circuits sampled from some Φ_m distribution whereby (a) the average length of a sampled circuit scales linearly with m, and (b) each circuit will deterministically output some n-bit string if implemented perfectly.

(ii) Process the data to obtain the average success probability (P_m) at each length m, meaning the probability that the expected bit-string was successfully output.

(iii) Fit P_m to $P_m = A + B\lambda^m$, and convert λ to an error rate via $r = \mathcal{N}(1 - \lambda)$ where $\mathcal{N}$ is a constant that we set to $\mathcal{N} = (4^n - 1)/4^n$ where n is the number of qubits.

Within this framework there are a range of different RB protocols, *e.g.*, those of Refs. [11–13], that vary in how the random circuits are chosen. Our methods can be used to upgrade any of these RB protocols to time-resolved techniques.

We focus on the non-intrusive approach to time-resolved RB, which is the following method:

1. Obtain data by rastering through the sampled circuits.

2. Use the general stability analysis to estimate the time-resolved success probability $p(t)$ for each circuit.

3. Average the time-resolved success probabilities for all circuits of the same RB length m to obtain time-resolved average success probabilities $P_m(t)$.

4. Apply the standard RB curve fitting analysis [step (iii) above] to $P_m(t_i)$ at every time of interest t_i.

The general instability analysis step in this process has some useful flexibility. It has two aspects that can be optimized to a particular task: the exact statistical hypothesis testing on the power spectra, and the selection of the frequencies to include in the time-resolved $p(t)$ models.

Because the RB circuits are random, they are broadly all sensitive to the same physical parameters, with the exact sensitivities depending on the precise details of the random circuit. So the frequencies in $p(t)$ will typically be the same in all the sampled circuits, even if the power in each frequency will generally vary between circuits. In this circumstance the averaged power spectrum is more powerful for detecting these frequencies, because the averaging suppresses measurement shot noise and amplifies signal if the same frequencies appear in most of the spectra. Moreover, here there is little penalty for erroneously including a frequency in the success probability trajectory in a few circuits — we are implementing a global analysis of the data, rather than trying to extract detailed diagnostic information. So we only perform hypothesis testing on the average power spectrum (*i.e.*, we set $w = 1$ in Supplementary Eqs. (22–23)), and then we include every statistically significant frequency in the model for each success probability.

I. Time-resolved RB simulation

In the main text we demonstrate time-resolved RB on simulated data. This demonstration is of time-resolved direct randomized benchmarking (DRB) [13] on two qubits, with a gate set consisting of a CPHASE gate between the qubits (*i.e.*, a controlled σ_z), rotations around the $\hat{x}$ and $\hat{y}$ axes by $\pm\frac{\pi}{2}$ and π on each qubit, and the Hadamard and idle gates on each qubit. The details of how DRB circuits are sampled (*i.e.*, the Φ_m of DRB) can be found in Ref. [13] — they are omitted here as they are not relevant to assessing the performance of time-resolved RB.

We simulated gates with time-dependent coherent phase errors and time-independent depolarizing errors. Each gate is modeled as the perfect gate followed by the error map

$$\mathcal{E}(t) = \mathcal{D}_\gamma \circ \mathcal{R}_z(\theta(t)), \tag{34}$$

on each qubit, where

$$\mathcal{D}_\gamma[\rho] = \gamma\rho + (1-\gamma)\mathbb{1}/2, \tag{35}$$

is a one-qubit depolarizing map, and

$$\mathcal{R}_z(\phi)[\rho] = \exp\left(-i\frac{\phi}{2}\sigma_z\right)\rho\exp\left(+i\frac{\phi}{2}\sigma_z\right). \tag{36}$$

We set $\gamma = 1 - 0.04/3$, making the probability of Pauli error 1%. The rotation angle is set to

$$\theta(t) = at + b\sin(\phi t) + c_t, \tag{37}$$

where $a = 2\pi\times 10^{-5}$, $b = 1.5\times 10^{-2}$, $\phi = 2\pi\times 10^{-2}$, and c_t is a Brownian stochastic process given by $c_0 = 0$ and $c_{t+1} = c_t + \mathcal{N}(0,\nu)$ where $\nu = 3/200$. The parameters in this error model have been chosen to give physically plausible phase trajectories, and to not be artificially easy for our methods (*e.g.*, they do not induce $p(t)$ that are exceptionally sparse in the Type-II DCT basis). They have no further significance. The time t starts at 0 and increases by 1 after a single run of any circuit.

In Fig. 2A of the main text the estimated time-resolved error rate is compared to the true time-dependent $r(t)$. The value of $r(t)$ is obtained by fitting the exact instantaneous average success probabilities for the set of 100 sampled circuits. This $r(t)$ is the target that time-resolved DRB with this particular set of random circuits is aiming for — it does not correspond to the large-sample-limit error rate that finite-sampling DRB is estimating. We choose this comparison because analysis of time-series data from one particular set of K randomly sampled circuits cannot correct for any finite sampling error introduced when sampling this set of circuits. By defining $r(t)$ in this way, we avoid conflating this finite-circuit-sampling error with any inaccuracies that are intrinsic to time-resolved benchmarking.

J. Gate set tomography

GST is a method for jointly reconstructing a set of gates, a state preparation and a measurement [14, 15]. GST is predicated on the standard Markovian model for errors in QIPs, whereby the prepared state is a fixed density operator, the gates are fixed completely positive and trace preserving (CPTP) maps — which are the linear transformations that map density operators to density operators — and the measurement is a fixed positive-operator valued measure. For our implementation of GST, we parameterize a CPTP gate by $\mathsf{G} = \Lambda \circ \mathsf{G}_{\mathsf{target}}$ where $\mathsf{G}_{\mathsf{target}}$ is the fixed target map and Λ is a CPTP map encompassing the errors in the gate. This error map is then parameterized as $\Lambda = \exp(G)$ with G defined by

$$G[\rho] = \sum_k \theta_k H_k[\rho] + \sum_{j,k} s_{jk} S_{jk}[\rho], \tag{38}$$

where the summations are over x, y and z, and

$$H_k[\rho] = \frac{i}{2}(\rho\sigma_k - \sigma_k\rho), \tag{39}$$

$$S_{jk}[\rho] = \sigma_j\rho\sigma_k - \frac{1}{2}(\rho\sigma_k\sigma_j + \sigma_k\sigma_j\rho). \tag{40}$$

Here, σ_x, σ_y and σ_z are the Pauli operators. The $\{H_k\}$ maps generate all unitary errors. If $(\theta_x, \theta_y, \theta_z) = \phi\hat{v}$ where $\hat{v}$ is a unit vector then the generated unitary is a rotation around the $\hat{v}$ axis of the Bloch sphere by an angle ϕ. So θ_x, θ_y and θ_z are the components of the unitary error along the $\hat{x}$, $\hat{y}$ and $\hat{z}$ axes in the standard sense. The $\{S_{ij}\}$ maps generate all non-unitary Markovian errors, so the matrix s dictates the stochastic error rates and the size of any amplitude damping errors. In this parameterization, the CPTP constraint is that s is positive semi-definite. Finally, note that this parameterization of Λ includes only infinitesimally-generated CPTP maps; it does not strictly permit every possible CPTP map.

K. Time-resolved GST simulation

In the main text, time-resolved GST is demonstrated on simulated data. Here we detail the error model that was simulated, and use this example to explain how we apply our methods to implement time-resolved GST. The simulation is for GST circuits out to $L = 128$ containing the gates $\mathsf{G_i}$, $\mathsf{G_x}$ and $\mathsf{G_y}$ with an error model that, when expressed in the parameterization above, consists of:

(i) Setting the s matrix for all three gates so that if there are no coherent errors Λ is a uniform depolarizing channel with a 1% error rate (this is $s \propto \mathbb{1}$).

(ii) Setting all the coherent error parameters to zero, except

$$\theta_z(\mathsf{G_i}) = \frac{2\pi}{10^3}\left[f_1(t) + f_2(t)\right],$$

$$\theta_x(\mathsf{G_x}) = \theta_y(\mathsf{G_y}) = \frac{4\pi}{10^3}\left[f_1(t) - f_2(t) + 0.5 f_{100}(t)\right],$$

where $\theta_k(\mathsf{G})$ is the H_k error rate for gate G, and $f_k(t)$ is the kth basis function of the Type-II DCT.

Time starts at 0 and increments by 1 after each raster. The trajectories of the time-dependent parameters over the 1000 simulated rasters are shown in Fig. 2D of the main text (and are also included in Fig 2C).

To implement time-resolved GST on this simulated data we first apply the general stability analysis. The resulting

$$\lambda_{\mathsf{p}} = -\log_{10}(\mathsf{p}), \tag{41}$$

statistic for every circuit is shown in Supplementary Fig. 2A, where p is the p-value of the largest peak in the power spectrum for that circuit. (Due to numerical accuracy limitations, the minimal p-value is $\mathsf{p} = 10^{-16}$). The value of λ_{p} varies strongly between circuits of the same approximate length L. The strong variations in λ_{p} imply that the time-varying errors are coherent, as stochastic errors have a fairly uniform impact across the GST circuits. Moreover, by inspecting the values of λ_{p} for the simple germs $\mathsf{G_i}$, $\mathsf{G_x}$ and $\mathsf{G_y}$ it becomes clear that $\theta_z(\mathsf{G_i})$, $\theta_x(\mathsf{G_x})$ and $\theta_y(\mathsf{G_y})$ are varying. For example, with the $\mathsf{G_i}$ germ it is the generalized Ramsey circuits for which λ_{p} is statistically significant, as highlighted in Supplementary Fig. 2B. These circuits amplify $\theta_z(\mathsf{G_i})$, so this parameter is likely varying over time. Similarly, it is generalized Rabi sequences that exhibit statistically significant λ_{p} for

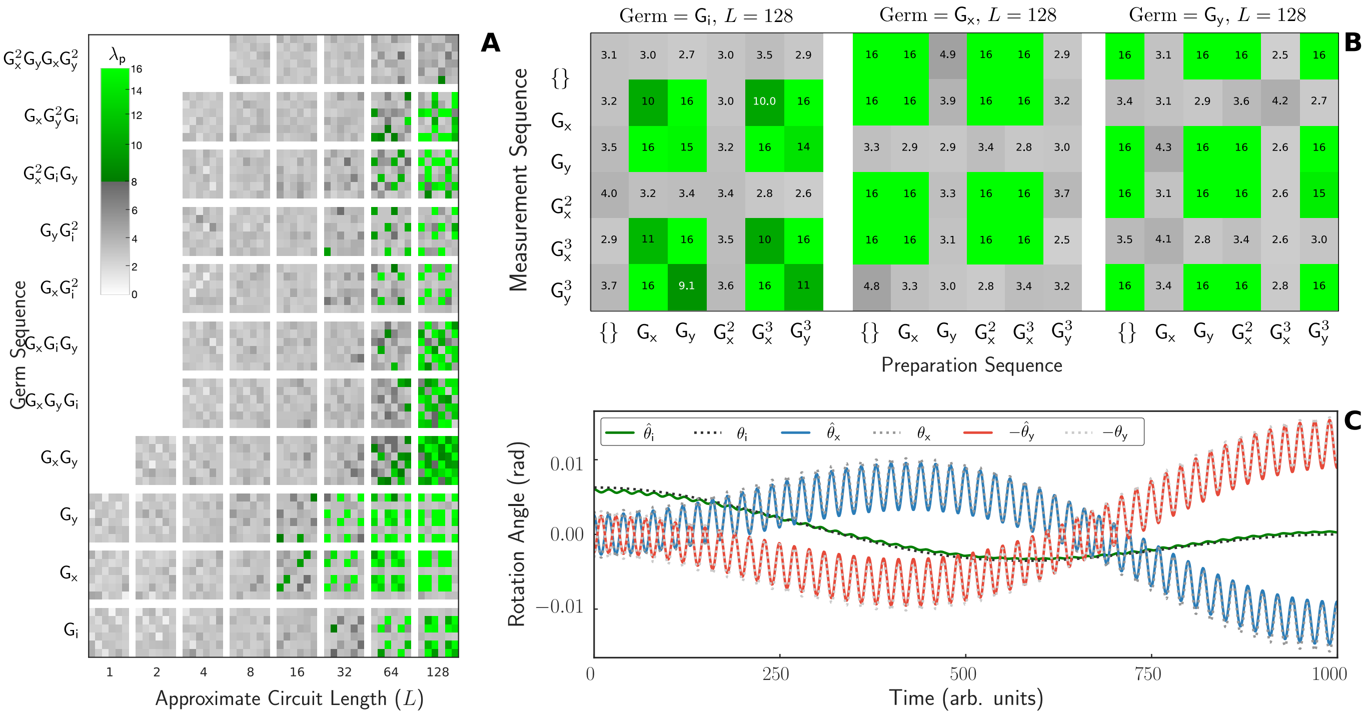

Supplementary Figure 2. **Model selection in time-resolved GST on simulated data**. **A-B.** The evidence for instability in the outcomes of each circuit in simulated GST data, as quantified by the test statistic λ_p. A pixel is colored when λ_p is large enough to be statistically significant, otherwise it is greyscale. The arrangement of the data is as in Fig. 3 of the main text. The pattern of significant λ_p is used to decide which model parameters to expand into a sum of Fourier coefficients. **C.** The results of time-resolved GST when we select a time-resolved parameterized model using a simple frequency selection technique that erroneously includes high-frequency components in the $\hat{z}$-axis rotation angle of G_i. The parameter estimates still closely track the true values, demonstrating that time-resolved GST can be successful even with simple model selection methods.

the G_x and G_y germs, and these circuits amplify over/under-rotation errors in these gates, *i.e.*, the parameters $\theta_x(G_x)$ and $\theta_y(G_y)$. So we conclude that $\theta_z(G_i)$, $\theta_x(G_x)$ and $\theta_y(G_y)$ should be expanded into a summation of Fourier coefficients. Having decided on the set of parameters that are to be expanded into a time-dependent functions, we now use the short-hand $\theta_i \equiv \theta_z(G_i)$, $\theta_x \equiv \theta_x(G_x)$ and $\theta_y \equiv \theta_y(G_y)$.

Deciding on the frequencies to include in the expansion of each of these parameters is more involved. One simple option is to use the frequencies that are statistically significant in the global power spectrum — the set $\mathbb{W} = \{1, 2, 3, 4, 97, 98, 99, 100, 101, 102\}$ — as the frequency set for all three parameters. This parameterized model ($\mathcal{M}_1$) includes all of the frequencies in the true model, but also sums and differences of these frequencies, as well as high-frequencies in the expansion of G_i that are not present in the true model. Yet, implementing MLE over this parameterized model still provides an accurate time-resolved estimate of θ_i, θ_x and θ_y, as shown in Supplementary Fig. 2C.

A more sophisticated model selection strategy is to select the frequencies to include in the expansion for θ_i (the set $\mathbb{W}_i$) to be those frequencies that are statistically significant in the circuits in which G_i is a germ, and similarly for θ_x and θ_y. This results in the sets $\mathbb{W}_i = \{1, 2, 3\}$, $\mathbb{W}_x = \{1, 2, 3, 100, 103\}$ and $\mathbb{W}_y = \{1, 2, 3, 5, 97, 100, 101, 103\}$, and constitutes a smaller parameterized model ($\mathcal{M}_2$). Even this smaller model still contains frequencies that are not in the true model, but each frequency set now only contains sums and differences of the frequencies that appear in the corresponding parameter. The MLE over this parameterized model is what is reported in the main text. The difference in the AIC scores of these two candidate parameterized models is $\mathrm{AIC}(\mathcal{M}_1) - \mathrm{AIC}(\mathcal{M}_2) \gg 1$. So the AIC favors discarding the erroneous parameters in $\mathcal{M}_1$.

L. Time-resolved GST on experimental data

We now detail the exact approach used to implement time-resolved GST on the data from the first of our two GST experiments (as discussed in the main text, we implemented standard time-independent GST on the data from the second experiment). As discussed in the main text, we use the λ_p statistics to motivate only expanding the $\hat{z}$-axis rotation angle of the G_i gate — *i.e.*, using the same notation as above, the parameter $\theta_z \equiv \theta_z(G_i)$ — into a sum of Fourier components. Specifically, we expand θ_z into

$$\theta_z = \gamma_0 + \sum_{\omega \in \mathbb{W}} \gamma_\omega f_\omega(t), \qquad (42)$$

where $\mathbb{W} = \{1, 2, 5, 7, 15\}$ is the set of significant frequencies in the average power spectrum (shown in Fig. 3D). This

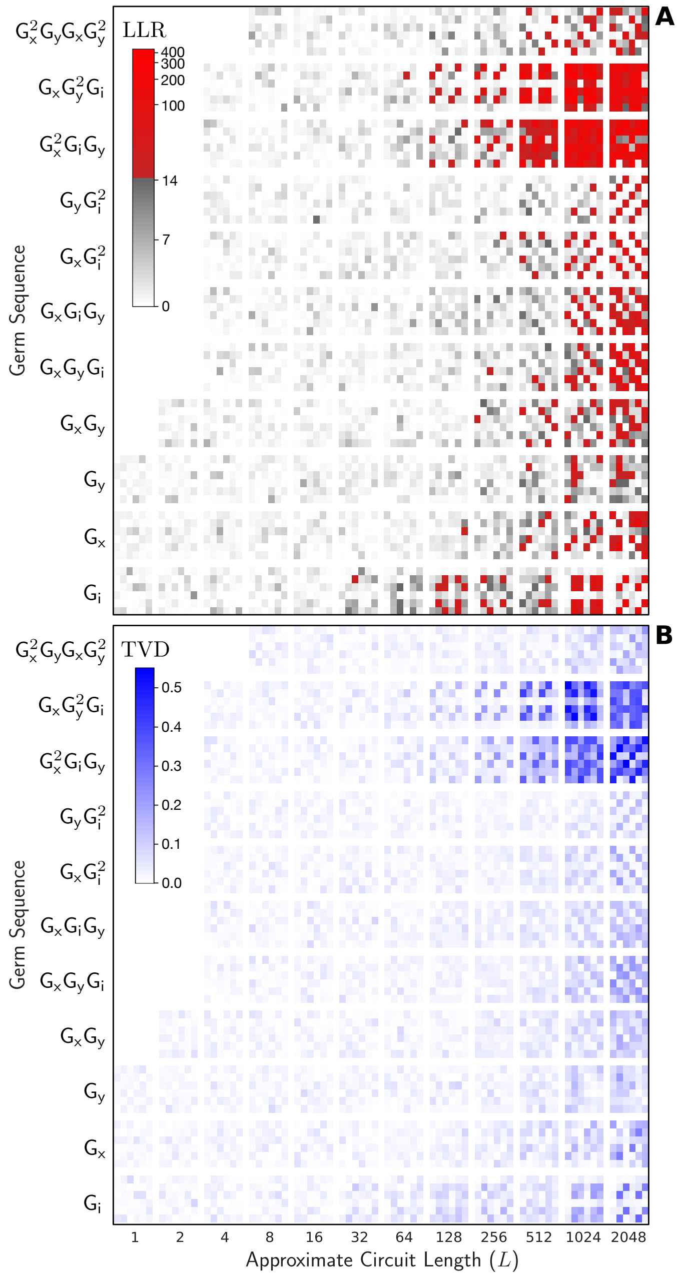

Supplementary Figure 3. **Model violation in standard GST on experimental data**. The discrepancy between the predictions of the time-independent GST estimate and the data, for the first GST experiment. **A.** The log-likelihood ratio (LLR) statistics of the GST estimate. A pixel is colored if and only if the LLR for that circuit is so large that it is inconsistent with the model of standard GST, at a global significance of 5%. **B.** The total variational distance (TVD) between the probabilities predicted by the GST estimate and the observed frequencies. The TVD is not being used as a statistic in a hypothesis test and so there is no differentiation between significant and insignificant TVDs.

method for the frequency selection is motivated by noting that, as we have decided that only θ_z exhibits time-dependence, all the frequencies in the averaged power spectrum must be attributable to variations in θ_z. So far this is exactly the same type of analysis as with the time-resolved GST on simulated data. However, fitting the model requires more care with experimental data.

Error rate	$\epsilon_\diamond \times 10^3$		$\epsilon_f \times 10^3$		$\epsilon_{spec,f} \times 10^3$	
Exp. #	1	2	1	2	1	2
G_i	3.45	0.40	0.02	0.07	0.02	0.07
G_x	1.25	0.38	0.03	0.06	0.03	0.06
G_y	0.82	0.53	0.15	0.04	0.15	0.04

Supplementary Table 1. **Estimated gate error rates**. Error rates for the three gates in the two GST experiments, obtained using time-resolved GST. In the first experiment the error rates of the G_i gate vary over time (see Fig. 3C of the main text). The values reported here are the average over the experiment duration. All other error rates are static over the course of each experiment. See the text for definitions of these three different error rates.

First we implemented standard time-independent GST on all the data. The predictions of this GST estimate are compared to the aggregated counts data in Supplementary Fig. 3. This summarizes the discrepancy between the data and the predictions of the model estimated by standard GST using (A) the log-likelihood ratio (LLR) and (B) the total variational distance (TVD). The LLR test statistic for a circuit is

$$\mathsf{LLR} = -2\log\left(\mathcal{L}_{\mathsf{gst}}/\mathcal{L}_{\mathsf{freq}}\right). \tag{43}$$

Here $\mathcal{L}_{gst}$ is the likelihood, given the data, of the outcome probabilities for that circuit that are predicted by the GST estimate. Conversely, $\mathcal{L}_{freq}$ is the likelihood of the outcome probabilities that are equal to the observed frequencies (*i.e.*, for each outcome, set the corresponding probability equal to the number of times that particular outcome was observed divided by the total number of outcome counts for the circuit). Under the null hypothesis that the model of standard GST is true, Wilks' theorem tells us that the LLR is asymptotically χ_1^2 distributed. This is because, since we have many circuits, the degrees of freedom per circuit is approximately zero for the GST model (used to compute $\mathcal{L}_{gst}$) and one for the model maximized over to compute $\mathcal{L}_{freq}$.The TVD for a circuit is

$$\mathsf{TVD} = \left| p_{\mathsf{model}} - \frac{1}{N}\sum_i x_i \right|, \tag{44}$$

where p_{model} is the probability for the circuit to output 1 predicted by the estimated model and $\mathbf{x}$ is the clickstream for the circuit. We do not use the TVD as a test static (it has less convenient properties for hypothesis testing than the LLR), but the TVD is useful as — unlike the LLR — it quantifies the distance between the observed frequencies and the predicted probabilities. There are statistically significant LLRs in Supplementary Fig. 3A, so the data are inconsistent with the model of standard GST.

From the general instability analysis, we know that there is some instability in the qubit (see Fig. 3A of the main text).

So we did not expect the time-independent model of standard GST to be consistent with the data. But, if drift was the only effect outside of GST's model that is needed to explain the data, we would expect the predictions of the estimated GST model to be inconsistent with the data only for those circuits that exhibit drift. This is not the case; comparing Supplementary Fig. 3 to Fig. 3A of the main text shows that the inconsistency between the estimate of standard GST and the data cannot all be attributed to detectable drift. In particular, the data from many of the long circuits containing germs other than $\mathsf{G_i}$ are irreconcilable with the model of GST, but there is no evidence for drift in these circuits. So there is some other non-Markovian effect that our time-resolved parameterized model does not take into account.

This sort of "model violation" interferes with the model selection and data fitting techniques that we rely on. If we fit our time-resolved parameterized model to all the data, the data that are inconsistent with any set of parameter values can pollute the estimates and render them meaningless. So we first estimate most of the time-independent parameters in the model — specifically, the state preparation, measurement, $\mathsf{G_x}$, $\mathsf{G_y}$ and the non-unitary errors matrix s for $\mathsf{G_i}$ — by implementing ordinary GST on the data from circuits with $L \leq 64$ (these circuits are short enough for the model of GST to be reasonably consistent with the data). Only the two time-independent coherent error parameters in $\mathsf{G_i}$ and θ_z — which is a summation of 6 Fourier coefficients — are not fixed by this initial fit. Using MLE, we then fit this 8-parameter time-resolved model to the time-series data for all circuits with $L \leq 64$ and all the circuits where $\mathsf{G_i}$ is a germ with $L \leq 1024$. This allows us to extract information about the time-dependent parameter without polluting the results with data that does not fit this model.

M. Estimated gate error rates

In the main text, we report the diamond distance error rate ($\epsilon_\diamond$) of the estimated (time-resolved) gate process matrices in the two GST experiments. Table 1 includes additional information on the gate error rates. In addition to $\epsilon_\diamond$, this table reports the entanglement infidelity (ϵ_f) and spectral entanglement infidelity ($\epsilon_{\mathsf{spec,f}}$) for each gate. The diamond distance error rate for process matrix G is

$$\epsilon_\diamond(\mathsf{G}, \mathsf{G}_{\text{target}}) = \frac{1}{2}||\mathsf{G} - \mathsf{G}_{\text{target}}||_\diamond, \quad (45)$$

where $||\cdot||_\diamond$ is the diamond norm [16] and $\mathsf{G}_{\text{target}}$ is the target process matrix that G ideally should be. The entanglement infidelity is

$$\epsilon_\mathsf{f}(\mathsf{G}, \mathsf{G}_{\text{target}}) = 1 - \langle\varphi|\left(\mathbb{1} \otimes \mathsf{G}\mathsf{G}^\dagger_{\text{target}}\right)(\varphi)|\varphi\rangle, \quad (46)$$

where φ is any maximally entangled state [17]. Although the entanglement infidelity is defined with respect to a maximally entangled state, note that it is well-defined for one-qubit gates and it does not imply that any entangling operations are being performed. Another commonly used gate infidelity is the average gate infidelity ($\bar{\epsilon}$). The entanglement infidelity is related to the average gate infidelity via a linear rescaling. Specifically, $\epsilon_\mathsf{f} = (d+1)\bar{\epsilon}/2$ [17] where d is the dimension, so here $d = 2$. We use the entanglement infidelity because $\epsilon_\diamond = \epsilon_\mathsf{f}$ for depolarizing errors, so the difference between $\epsilon_\diamond$ and ϵ_f is a rough measure of how structured the errors are, *e.g.*, for coherent errors $\epsilon_\diamond > \epsilon_\mathsf{f}$.

The spectral entanglement infidelity ($\epsilon_{\mathsf{spec,f}}$) is defined to be

$$\epsilon_{\mathsf{spec,f}} = \epsilon_\mathsf{f}\left(\mathsf{Spec}(\mathsf{G}), \mathsf{Spec}(\mathsf{G}_{\text{target}})\right), \quad (47)$$

where $\mathsf{Spec}(\mathsf{A})$ is the diagonal matrix that has the eigenvalues of the matrix A on the diagonal (and the relative ordering of the eigenvalues in the two matrices is chosen to minimize $\epsilon_{\mathsf{spec,f}}$). The spectral entanglement infidelity is an alternative to the standard entanglement infidelity that is invariant under transformation of the "GST gauge" (see Ref. [14] for discussions of this gauge).

Looking at Table 1 we see that all three gates have a smaller diamond distance error rate in the second experiment, with a decrease from $\epsilon_\diamond \sim 0.5\%$ to $\epsilon_\diamond \sim 0.05\%$. The infidelity also decreases for $\mathsf{G_y}$, but, in contrast, for $\mathsf{G_i}$ and $\mathsf{G_x}$ the infidelity increases from $\epsilon_\mathsf{f} \sim 0.003\%$ to $\epsilon_\mathsf{f} \sim 0.006\%$. The diamond distance error rate is sensitive at order θ to a coherent error with a rotation angle of θ, whereas the infidelity is sensitive to coherent errors only at order θ^2. So, a decrease in $\epsilon_\diamond$ and an increase in ϵ_f is a sign that the magnitude of the coherent errors (*i.e.*, calibration errors) has been reduced in the second experiment, but that the rate of stochastic errors has slightly increased.

SUPPLEMENTARY NOTE 3 — EXPERIMENT DESIGN

In this section we discuss how to design experiments to efficiently detect and characterize instability. This includes details on the relationship between the amount of data taken and the sensitivity of our methods.

N. How much data do we need?

Consider looking for instability in a given set of circuits. For example, we could be running an algorithm, or syndrome extraction circuits, or the circuits from some benchmarking routine. Furthermore, assume that we are going to collect data by rastering through this set of circuits N times. It then only remains to choose N, the length of the clickstream for each circuit. In this circumstance, it is important to understand how the sensitivity of our methods varies with N.

Each circuit has some discrete-time probability trajectory $\mathbf{p} = (p_0, p_1, \ldots, p_{N-1})$. We can always express this as a summation over the basis vectors of F. As we use the Type-II DCT for all of our explicit data analysis, we will use this basis for clarity. In this basis, we write

$$p_i = \bar{p} + \sum_{\omega=1}^{N-1} \gamma_\omega \cos\left(\frac{\omega\pi}{N}\left(i + \frac{1}{2}\right)\right), \quad (48)$$

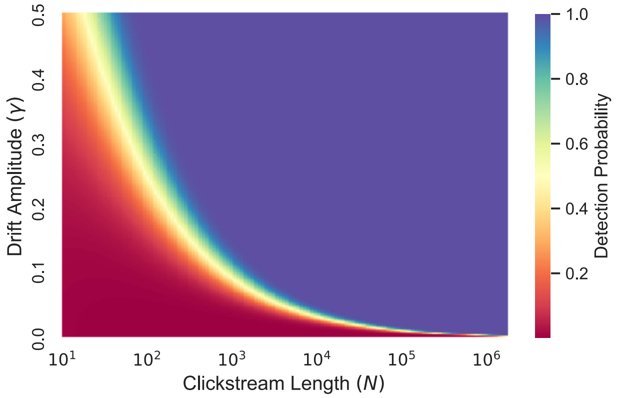

Supplementary Figure 4. **Instability detection sensitivity**. The probability of detecting that $\mathbf{p}$ has a nonzero component at ω, from a clickstream of length N and with an amplitude of γ at any non-zero basis frequency ω, using tests of the power spectrum at a statistical significance of 5%. Here γ corresponds to the amplitude in an unnormalized DCT expansion of $\mathbf{p}$ (*i.e.*, the expansion of Supplementary Eq. (48)), meaning that if this is the only non-zero frequency with a non-zero amplitude in this expansion of $\mathbf{p}$ then $\mathbf{p}$ oscillates between $\bar{p} \pm \gamma$.

for some $\{\gamma_\omega\}$ amplitudes. We can use our statistical model for the frequency-domain data (see earlier) to approximately calculate the probability of successfully detecting that some γ_ω is non-zero, as a function of γ_ω, N and the test significance α. In particular, the probability of detecting that $\mathbf{p}$ has a nonzero component at ω, from a clickstream of length N and with tests at a statistical significance of α, is approximately

$$P_{\rm det}(N, \gamma_\omega, \alpha) = 1 - \frac{1}{2}\left[\mathrm{erf}\left(\frac{\delta_+}{\sqrt{2}}\right) + \mathrm{erf}\left(\frac{\delta_-}{\sqrt{2}}\right)\right]. \quad (49)$$

Here, erf(·) is the error function,

$$\delta_\pm = \sqrt{T(\alpha, N)} \pm \gamma_\omega \sqrt{\frac{N}{2\bar{p}(1-\bar{p})}}, \quad (50)$$

and $T(\alpha, N)$ is the power significance threshold for the hypothesis test at that frequency. In the case of testing a single clickstream, and splitting the test significance equally over all $N-1$ non-zero frequencies (as we do for all our data analysis, but which is not necessarily always desirable), this is given by

$$T(\alpha, N) = \mathsf{CDF}_1^{-1}\left[1 - \frac{\alpha}{(N-1)}\right], \quad (51)$$

where CDF_k is the χ^2_k cumulative distribution function. If we are testing C clickstreams, we simply replace $\alpha \to \alpha/C$ in this equation.

Supplementary Fig. 4 shows a heatmap of $P_{\rm det}(N, \gamma, 0.05)$ as N and γ are varied. From this plot we can read off the clickstream length required to reliably detect an instability of a given amplitude. It shows that only moderate amounts of data are required to detect relatively small instabilities, *e.g.*, an instability of amplitude $\gamma \approx 0.1$ can be reliably detected with $N \approx 1000$. This quantity of data is routinely taken to estimate static circuit outcome distributions.

As well as detecting instabilities in probability trajectories, our methods also estimate $p(t)$. To quantify the accuracy of our probability trajectory estimation we use simulations. We simulated clickstream data sampled from a pure-tone probability trajectory $\mathbf{p}(\gamma)$:

$$p_i(\gamma) = 0.5 + \gamma \cos\left(\frac{\omega\pi}{N}\left(i + \frac{1}{2}\right)\right). \quad (52)$$

Supplementary Fig. 5 shows both the instability detection probability and the error in our estimator. Supplementary Fig. 5B shows the root mean squared error (RMSE) of our estimator for three values of γ (unbroken lines), alongside the corresponding baselines set by estimating $p(t)$ as a constant given by the mean of the clickstream (dotted lines). This is the mean RMSE, obtained in 10^5 simulations, and note that this simulation used the "Fourier filter" variant of our estimator (as it is faster to calculate than the MLE version). In the regime where N is so low that instability is almost never detected, the RMSE of our estimator is essentially equal to the RMSE estimator of the baseline clickstream-mean estimate. This is because our estimate is the mean of the clickstream unless instability is detected. In fact, when N is so low that instability is almost never detected (as shown in Supplementary Fig. 5A) our estimator actually performs worse than the baseline, on average. This is because in approximately 5% of instances (for testing at 5% statistical significance) our detection routine will spuriously detect that there is a component of $\mathbf{p}$ at some random frequency, and so we will spuriously add a component at that frequency into our estimator. Comparing Supplementary Fig. 5A and 5B, we see that the RMSE of our estimator rapidly improves once N moves into the regime where instability at frequency ω is successfully detected with high probability. Once the detection probability saturates at essentially 1, the RMSE then follows $O(1/\sqrt{N})$ scaling. This is because the RMSE in the estimate of each amplitude in our selected model is essentially shot-noise limited.

So far we have considered how long a single clickstream needs to be to reliably detect instabilities of a given magnitude. But our analysis can also jointly test the data from multiple circuits, by calculating and then testing an averaged power spectrum. This allows for much greater sensitivity for a fixed N, where N is the clickstream length for each circuit. Supplementary Fig. 6 demonstrates this enhancement, by plotting the minimum N required for a 50% detection probability ($N_{\rm min}$) when averaging spectra from 1, 10, 100 and 1000 circuits. This is for the case of clickstreams that are all sampled from the same pure-tone probability trajectory $\mathbf{p}(\gamma)$. This case is chosen as it provides the maximal enhancement from averaging spectra, and — although it is unlikely that multiple circuits will have identical $p(t)$ — there are many circumstances under which many or all of a set of circuits would contain drift at the same frequencies.

Supplementary Fig. 6 shows that increasing the number of circuits provides a substantial reduction in $N_{\rm min}$. For example, if $\gamma = 0.1$ then $N_{\rm min} \approx 100$ for 100 clickstreams whereas $N_{\rm min} \approx 1000$ for a single clickstream. But note that the sen-

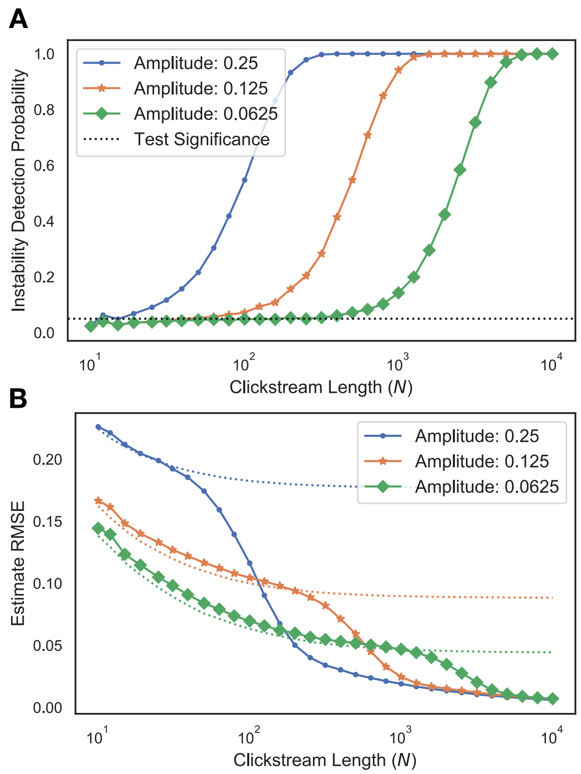

Supplementary Figure 5. **Detection probability and estimation error**. **A.** The detection probability versus clickstream length for data sampled from a pure-tone probability $p(t) = 0.5 + \gamma \cos(\omega t + \tau)$ for three values of the amplitude γ, where ω and τ are such that this is a pure-tone in the DCT basis. Note that this is the probability of detecting significant power at any non-zero ω, so the low-N limit is the false detection probability of approximately the test significance α, set here to 5%. **B.** The root mean squared error (RMSE) of our estimate of $p(t)$. The dotted lines show the RMSE of the constant-probability estimates, whereby we estimate $p(t)$ to be the mean of the clickstream. All quantities are calculated by averaging over the analysis results from 10^5 simulated clickstream instances.

sitivity enhancement from increasing the number of circuits is smaller than that from increasing the clickstream length. As illustrated in the above example, to obtain a 50% detection probability at $\gamma = 0.1$ we need 10 times more bits in total in the 100 clickstream case than in the single clickstream case. Essentially, this is due to the difference between the $O(1/\sqrt{C})$ shot-noise suppression obtained when averaging C spectra with independent statistical fluctuations verses the linear growth with N of the power in the spectrum of $\mathbf{p}$ (for $\mathbf{p}$ with an N-independent variance).

O. Choosing the circuit repetition rate

Our methods can be used for both dedicated drift characterization and for auxiliary data analysis. For dedicated drift characterization we need to carefully chose a set of quantum circuits that are as sensitive as possible to the specific parameters under study, as we discussed in the main text. Greater sensitivity results in large fluctuations in the circuits' outcome probabilities, for a given instability on the parameters of interest, meaning that fewer data are needed to reliably detect and characterize the drift — as quantified above (see Supplementary Fig. 4-6). But there is also another important aspect for designing dedicated drift characterization experiments: the rate that the circuits need to be repeated. If the dedicated drift characterization circuits can be run sufficiently infrequently then it becomes possible to interleave them with application circuits. Furthermore, understanding the effect of reducing the repetition rate is important even in experiments run only for the purpose of drift characterization — as we will often have to decide between running more circuits, or repeating fewer circuits at a faster rate. For example, in the case of both GST and Ramsey experiments, we must choose the maximum circuit length, and this directly impacts the rate at which we can raster through the full set of circuits.

Reducing the repetition rate of the drift characterization circuits — *i.e.*, increasing the time between each run of a circuit, t_{gap} — will have two effects. Firstly, for a fixed experiment duration we will obtain fewer data points, *i.e.*, the clickstreams will be shorter. The impact of the clickstream length on the sensitivity of our methods has already been discussed in detail earlier (see Supplementary Fig. 4-6). But note that, for dedicated drift characterization, we are free to choose the circuits that maximally amplify the parameters we expect might be drifting — so that the oscillations in the $p(t)$ need only be small if they are small in every possible circuit (under the assumption that we have correctly identified all the parameters that might be drifting). Therefore, relatively low N will typically still allow for sensitive drift characterization. In the main text we demonstrated this, by discarding 80% of our Ramsey data (giving $N = 1200$) and showing that we still obtain a high-precision time-resolved estimate of the qubit detuning (see Supplementary Fig. 1E). Moreover, much more of that data could be discarded if we only wanted to detect drift and obtain a rough time-resolved estimate of the detuning.

The second impact of increasing t_{gap} is that the sensitivity to high-frequency drift is reduced. As with all signal analysis techniques that use regularly sampled data, there is a Nyquist limit — *i.e.*, oscillations at a frequency of $1/2t_{\text{gap}}$ are undetectable, and oscillations at higher frequencies are either aliased, or they are undetectable if they are integer multiples of this frequency. The repetition rate should therefore be chosen to maintain sensitivity to the frequency range of interest. As illustrated by our analysis of the Ramsey experiment, this means that we can have a slow repetition rate if there is only low-frequency drift.

SUPPLEMENTARY REFERENCES

[1] K Ramamohan Rao and Ping Yip, *Discrete cosine transform: algorithms, advantages, applications* (Academic press, 2014).

[2] Nasir Ahmed, T. Natarajan, and Kamisetty R Rao, "Discrete cosine transform," IEEE Trans. Comput. **100**, 90–93 (1974).

[3] Frank A Feldman, "Fast spectral tests for measuring nonrandomness and the DES," in *Conference on the Theory and Appli-*

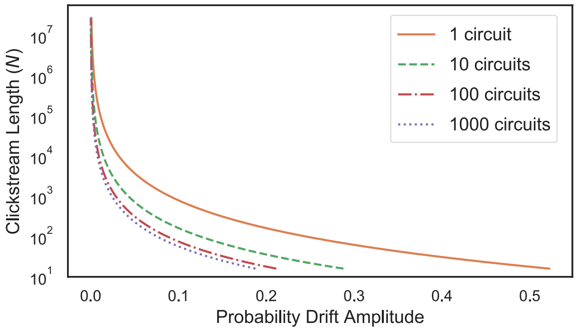

Supplementary Figure 6. **Detection probability with circuit number**. The minimum clickstream length N required for a 50% instability detection probability when averaging spectra from 1, 10, 100 and 1000 circuits. The clickstreams for all circuits are sampled from the same probability trajectory $p(t) = 0.5 + \gamma \cos(\omega t + \tau)$, where ω and τ are such that this is a pure-tone in the DCT basis.

cation of Cryptographic Techniques (Springer, 1987) pp. 243–254.

[4] M Zechmeister and M Kürster, "The generalised Lomb-Scargle periodogram – a new formalism for the floating-mean and keplerian periodograms," Astron. Astrophys. **496**, 577–584 (2009).

[5] Jacob T VanderPlas, "Understanding the Lomb–Scargle periodogram," Astro. J. Supplement Series **236**, 16 (2018).

[6] Erich L Lehmann and Joseph P Romano, *Testing statistical hypotheses* (Springer Science & Business Media, 2006).

[7] Juliet Popper Shaffer, "Multiple hypothesis testing," Annual review of psychology **46**, 561–584 (1995).

[8] Yoav Benjamini and Yosef Hochberg, "Controlling the false discovery rate: a practical and powerful approach to multiple testing," J. Roy. Statist. Soc. Ser. B , 289–300 (1995).

[9] Patrick Billingsley, *Probability and measure*, 3rd ed. (John Wiley & Sons, 1995).

[10] Hirotugu Akaike, "A new look at the statistical model identification," IEEE Trans. Autom. Control , 716–723 (1974).

[11] Emanuel Knill, D Leibfried, R Reichle, J Britton, RB Blakestad, JD Jost, C Langer, R Ozeri, S Seidelin, and DJ Wineland, "Randomized benchmarking of quantum gates," Phys. Rev. A **77**, 012307 (2008).

[12] Easwar Magesan, Jay M Gambetta, and Joseph Emerson, "Scalable and robust randomized benchmarking of quantum processes," Phys. Rev. Lett. **106**, 180504 (2011).

[13] Timothy J Proctor, Arnaud Carignan-Dugas, Kenneth Rudinger, Erik Nielsen, Robin Blume-Kohout, and Kevin Young, "Direct randomized benchmarking for multiqubit devices," Phys. Rev. Lett. **123** (2019).

[14] Robin Blume-Kohout, John King Gamble, Erik Nielsen, Kenneth Rudinger, Jonathan Mizrahi, Kevin Fortier, and Peter Maunz, "Demonstration of qubit operations below a rigorous fault tolerance threshold with gate set tomography," Nat. Commun. **8**, 14485 (2017).

[15] Seth T Merkel, Jay M Gambetta, John A Smolin, Stefano Poletto, Antonio D Córcoles, Blake R Johnson, Colm A Ryan, and Matthias Steffen, "Self-consistent quantum process tomography," Phys. Rev. A **87**, 062119 (2013).

[16] John Watrous, "Notes on super-operator norms induced by schatten norms," Quantum Information & Computation **5**, 58–68 (2005).

[17] Michael A Nielsen, "A simple formula for the average gate fidelity of a quantum dynamical operation," Phys. Lett. A **303**, 249–252 (2002).
